# FlyPredictome: A structural atlas of predicted protein-protein interactions in *Drosophila*

**DOI:** 10.64898/2026.04.14.718529

**Published:** 2026-05-11

**Authors:** Ah-Ram Kim, Aram Comjean, Austin Veal, Jonathan Rodiger, Myeonghoon Han, Yanhui Hu, Norbert Perrimon

**Affiliations:** 1 Department of Genetics, Blavatnik Institute, Harvard Medical School, Boston, MA, USA; 2 Howard Hughes Medical Institute, Harvard Medical School, Boston, MA, USA

## Abstract

Protein-protein interactions (PPIs) are fundamental to cellular function. Yet most *Drosophila* PPIs remain structurally uncharacterized despite the wealth of genetic and biochemical data available for this organism. Here we present FlyPredictome, a structural interactome based on 1.5 million pairwise AlphaFold-Multimer predictions. Using a local confidence metric that performs robustly for interactions involving flexible and disordered proteins, we systematically assess experimentally reported *Drosophila* PPIs and predict direct binding interfaces at residue-level resolution. Testing their functional relevance, we find that phenotype-associated missense mutations are enriched at predicted interaction interfaces. Building on these validated predictions, we construct an evidence-supported PPI network, revealing modular organization from signaling pathways to individual protein complexes. FlyPredictome is available as an open database, providing a structural foundation for interaction discovery in *Drosophila*.

## Introduction

*Drosophila melanogaster* has been a foundational model for dissecting fundamental biological mechanisms, including cell signaling and development. Forward and reverse genetic screens have mapped the core components and epistatic hierarchies of conserved pathways, including Notch, Wnt/Wingless, Hedgehog, JAK-STAT, Hippo, Toll, and receptor tyrosine kinase cascades, establishing a rich catalog of functional relationships among signaling proteins (reviewed in St Johnston, 2002; Bier, 2005; Perrimon et al., 2012; Attrill et al., 2024). Yet genetic interaction does not equate to physical interaction: a suppressor or enhancer relationship establishes functional linkage but does not reveal whether two proteins bind directly, which domains mediate the contact, or which residues form the interface. Even for most genetically defined pathway connections, the structural basis of the underlying physical interaction remains largely unknown.

Large-scale experimental approaches have expanded the catalog of physical interactions. The FlyBi yeast two-hybrid screen tested >10,000 *Drosophila* proteins in all-by-all screens, identifying ~8,700 binary interactions (Tang et al., 2023), while affinity purification–mass spectrometry (AP-MS) studies (DPiM, DPiM2) mapped co-complex associations across thousands of baits (Guruharsha et al., 2011; Bhat et al., 2024). However, these datasets remain incomplete in coverage and provide no structural information about the interaction interface. Recent advances in deep learning-based structure prediction, including AlphaFold (Jumper et al., 2021) and RoseTTAFold (Baek et al., 2021), have enabled prediction of three-dimensional structures of protein complexes. These methods have been applied at proteome scale to generate structural interactomes across multiple organisms (Humphreys et al., 2021; Burke et al., 2023; Schmid and Walter, 2025; Schmid et al., 2025; Zhang et al., 2025; Han et al., 2026; Qi et al., 2026). Although structural predictions for *Drosophila* protein subsets have been reported (Kawaguchi et al., 2025; Peng and Zhao, 2026), a proteome-scale structural interactome has not yet been generated for *Drosophila*.

Here we present FlyPredictome, a structural atlas of the *Drosophila* protein interactome based on 1.5 million pairwise AlphaFold-Multimer predictions. We score predictions using a local confidence metric (integrated Local Interaction Score, iLIS), evaluate recovery of experimentally reported *Drosophila* PPIs, and provide structural models at residue-level resolution. We further test predicted interfaces by demonstrating that phenotype-associated missense mutations are enriched at predicted interaction sites. We organize evidence-supported predictions into a hierarchically clustered PPI network of over 21,000 interactions, revealing functional organization from pathway-level modules down to individual protein complexes. Finally, we provide the FlyPredictome database and interactive network viewer as open resources for the community.

## Results

### iLIS: a local confidence metric for identifying protein-protein interactions

**Figure 1.**
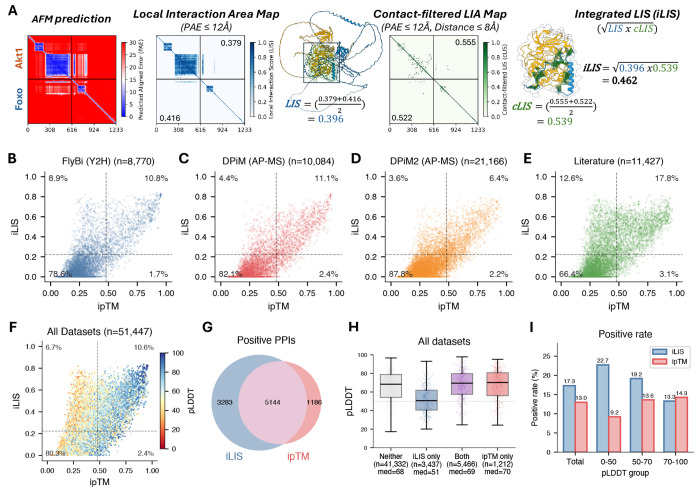
iLIS benchmarking and evaluation against experimental PPI datasets. **(A)** Schematic of iLIS computation. An AFM-predicted complex is scored by extracting the Local Interaction Area (LIA; PAE ≤ 12 Å), computing LIS (domain-level confidence) and cLIS (contact-filtered confidence at Cβ-Cβ ≤ 8 Å), and combining them as iLIS = √(LIS × cLIS). Example shown: Akt1–Foxo. **(B–E)** iLIS versus ipTM scatter plots for *Drosophila* PPIs from (B) FlyBi Y2H, (C) DPiM AP-MS, (D) DPiM2 AP-MS, and (E) literature-curated datasets. Dashed lines indicate 10% FPR thresholds for iLIS (≥ 0.223) and ipTM (≥ 0.48). Percentages in each quadrant indicate the fraction of experimentally reported interactions classified as positive by each metric. **(F)** Combined scatter plot across all datasets, colored by pLDDT. Low-pLDDT interactions (warm colors) are preferentially rescued by iLIS. **(G)** Venn diagram showing overlap between iLIS-positive and ipTM-positive PPIs. iLIS uniquely identifies 3,283 interactions missed by ipTM. **(H)** pLDDT score distribution for PPIs classified by each metric. iLIS-only PPIs (median pLDDT = 51) are significantly enriched for flexible/disordered proteins compared to the shared set (median = 69) or ipTM-only set (median = 70). **(I)** Positive prediction rate by pLDDT subgroup for iLIS versus ipTM. The largest difference is at pLDDT 0–50, where iLIS achieves 22.7% compared to 9.2% for ipTM.

To evaluate the ability of AlphaFold-Multimer (AFM; Evans et al., 2021) to predict protein-protein interactions (PPIs), we tested it on Positive Reference Sets (PRS) from proteome-scale yeast (Yu et al., 2008), fly (Tang et al., 2023), and human (Braun et al., 2009) Y2H screens. Several well-established PPIs, including p53–MDM2 (human), Emc–Da (fly), and SEC9–SSO1 (yeast), received low scores from ipTM — a widely used metric for evaluating AFM prediction confidence — despite forming clearly defined local interfaces visible in the Predicted Aligned Error (PAE) heatmaps (Figure S1.1A). We therefore developed iLIS, a local confidence metric building on the LIS framework (Kim et al., 2024). iLIS is the geometric mean of two component scores: LIS, which scores the confident interaction domain (PAE ≤ 12 Å), and cLIS, which further restricts scoring to residues in direct intermolecular contact (Cβ-Cβ ≤ 8 Å within the PAE ≤ 12 Å domain). This formulation integrates both domain-level and contact-level confidence into a single score (Figure 1A).

We benchmarked iLIS against multiple confidence metrics using 774 positive PPIs (from yeast, fly, and human Y2H PRS and structure-validated interactions from the ELM database; Kumar et al., 2024) and 6,004 controls (Random Reference Sets [RRS], GFP controls, and Wingless [Wg] subcellular compartment controls; see Methods for confidence metrics and benchmark dataset).

Spearman correlation analysis revealed distinct clustering among metrics, with iLIS showing the highest correlation with positive PPIs (Figure S1.2A–B). Local confidence metrics (including iLIS, ipSAE, and actifpTM) consistently outperformed other metrics such as ipTM and pDockQ across all pLDDT subgroups in ROC analysis (Figure S1.2C–D), with particularly clear separation for low-pLDDT proteins, characteristic of flexible or intrinsically disordered regions (Figure S1.2E–F). iLIS achieved strong statistical separation between positive and control PPIs across all pLDDT subgroups (Figure S1.2G–H; bootstrap benchmarking results for all metrics in Table S1). iLIS also maintained robust performance on a time-split benchmark using structures deposited after the AFM training data cutoff (Figure S1.2I–J). To enable downstream PPI classification at large scale, we established thresholds at 10% false positive rate (FPR) for iLIS (≥ 0.223) and ipTM (≥ 0.48) (Table S2). At these thresholds, iLIS rescued 47 well-established PPIs from the PRS that fell below the ipTM threshold (Figure S1.1B–C), including all low-pLDDT examples shown in Figure S1.1A.

### Benchmarking iLIS across experimental and computational PPI datasets

We next evaluated predictions against large-scale *Drosophila* experimental PPI datasets, including the FlyBi Y2H screen (Tang et al., 2023) and the DPiM/DPiM2 AP-MS interactomes (Guruharsha et al., 2011; Bhat et al., 2024). We recovered approximately 20% of FlyBi, 16% of DPiM, and 10% of DPiM2 interactions as positive (iLIS ≥ 0.223) (Figure 1B–D). The higher recovery of Y2H interactions compared to AP-MS may reflect that Y2H detects direct binary contacts more amenable to structural prediction, whereas AP-MS co-complex associations often include indirect interactions mediated by additional subunits. Unrecovered pairs may include indirect interactions through bridging factors, transient contacts that do not form persistent interfaces, or false positives inherent to high-throughput screens.

We further assessed recovery of a comprehensive set of literature-reported *Drosophila* PPIs sourced from FlyBase (Öztürk-Çolak et al., 2024), BioGRID (Oughtred et al., 2021), and MIST (Hu et al., 2018). Of these curated PPIs, ~30% were positively predicted (Figure 1E). This higher recovery rate compared to the large-scale proteomics screens likely reflects the nature of literature-curated interactions, which are typically validated through focused biochemical studies in individual laboratories and enriched for stable, reproducible complexes. Across all datasets, iLIS consistently identified positive PPIs that scored below the ipTM threshold (Figure 1B–F). When comparing the two metrics directly, iLIS identified thousands of additional interactions that fell below the ipTM threshold (Figure 1G), and these iLIS-only PPIs featured significantly lower pLDDT scores (Figure 1F, H), confirming that iLIS preferentially identifies interactions involving flexible proteins. This advantage was most pronounced at low pLDDT (0–50), where iLIS achieved a 22.7% positive rate compared to 9.2% for ipTM (Figure 1I).

We extended this analysis to two recent computational PPI prediction datasets. A recent *Drosophila* set of 27,711 AlphaFold-Multimer predictions (Peng and Zhao, 2026; 27,683 unique gene pairs after collapsing isoform duplicates), drawn from STRING ≥ 0.9, was primarily scored with pDockQ, which underperformed iLIS in our benchmark (Figure S1.2). On these predictions, iLIS (≥ 0.223) classified 6,671 pairs (24%) as positive, of which 1,070 were missed by pDockQ (< 0.23) but were independently flagged by Peng and Zhao as “possible” interactions based on inter-chain PAE ≤ 10 Å (Figure S1.3A,B); the two metrics make partly non-overlapping calls. A recent human predictome (Schmid et al., 2025) provides AlphaFold-Multimer predictions for 1,614,047 protein pairs nominated by the Knowledge-Integrated Rapid Classifier (KIRC), a random forest trained on 47 prior-knowledge biological features. From this dataset, iLIS (≥ 0.223) classified 227,714 pairs (14.1%) as positive (Figure S1.3C; Table S9), recovering 99.5% (16,480 of 16,557) of the high-confidence pairs identified by their SPOC classifier (SPOC ≥ 0.86). Together, these analyses indicate that iLIS is applicable across organisms and prediction datasets.

### Predicted structural models across diverse *Drosophila* pathways

**Figure 2.**
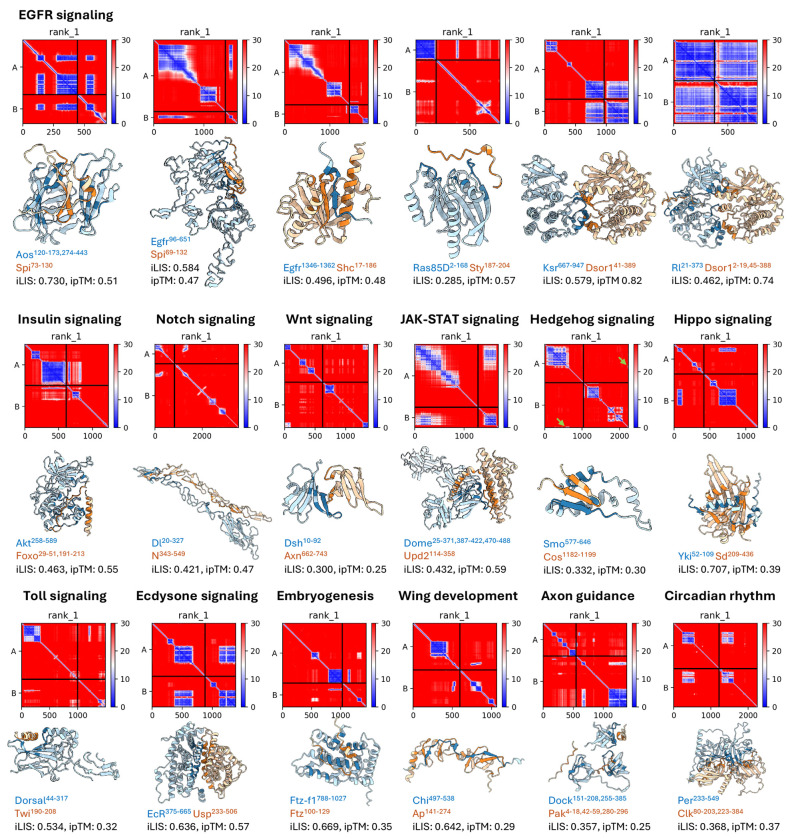
Predicted structural models of major signaling pathway PPIs. AFM-predicted structural complexes along the EGFR/Ras-MAPK cascade (top row) and across 12 additional signaling contexts (middle and bottom rows). For each pair, the Predicted Aligned Error (PAE) heatmap (top) and predicted 3D structure (bottom) are shown. In the PAE heatmaps, each cell represents the expected positional error (in Å) between two residues; low values (dark blue) indicate high-confidence relative positioning, while off-diagonal blue blocks between the two chains mark the predicted interaction interface. In the structures, chain A is colored in blue and chain B in orange, indicating Local Interaction Residue (LIR), with darker shading indicating contact LIR (cLIR). Residue ranges of the interacting domains, iLIS, and ipTM scores are indicated below each structure; only rank 1 predictions are shown. Green arrows highlight small interaction interfaces (in Smo–Cos). Some complexes, such as Dsh–Axn (iLIS = 0.300, ipTM = 0.25) and Smo–Cos (iLIS = 0.332, ipTM = 0.30), have low ipTM scores despite forming high-confidence local interfaces (high iLIS), illustrating the advantage of local metrics for interactions mediated by flexible regions.

Beyond metric benchmarking and recovery analysis, we asked whether these structural models could predict the direct physical interactions that underlie established signaling pathways in which the pathway components are well defined genetically but the structural basis of many individual binding events remains unknown. As a representative example, Figure 2 illustrates the predicted structural complexes along the Epidermal Growth Factor Receptor (EGFR)/Ras-MAPK cascade, one of the best-characterized receptor tyrosine kinase pathways in *Drosophila* (reviewed in Sopko and Perrimon, 2013; Shilo, 2014). Our predictions provide structural models for direct PPIs throughout the cascade, from ligand regulation (Argos–Spitz) and receptor activation (Egfr–Spitz) to adaptor recruitment (Egfr–Shc), feedback regulation (Ras85D–Sprouty), scaffold assembly (KSR–Dsor1), and the terminal MAPK relay (Rolled–Dsor1).

This predictive capability extends well beyond a single signaling cascade. Figure 2 further catalogs predicted complexes across 12 additional biological contexts spanning major signaling pathways, developmental processes, and behavioral programs. Notably, Chip binds Apterous LIM domains (Morcillo et al., 1997), and GST pulldown with deletion constructs mapped this interaction to the C-terminal LIM Interaction Domain (LID) of Chip (Torigoi et al., 2000); our predicted Chip–Apterous interface coincides with this experimentally defined region, illustrating that iLIS can recover interfaces mapped through biochemical assays. Together, these examples demonstrate that our predictions provide structural models of direct PPIs across diverse *Drosophila* pathways.

### Phenotype-associated alleles are enriched at predicted interfaces

Next, to assess whether the predicted interfaces are functionally important, we examined whether missense alleles (point mutations associated with phenotypic effects identified through genetic studies) are preferentially associated with predicted interaction sites. Missense mutations were compiled from FlyBase allele descriptions and genetic interaction records, and each was mapped to the corresponding residue in AFM-predicted structural models (see Methods). After filtering for validated amino acid identity at the mapped position, 2,761 unique mutations across 995 genes were retained for analysis. For each residue in a protein, we computed two interaction frequency metrics: the LIR (Local Interaction Residue) percentage, which measures how often that residue falls within a confident interaction domain (PAE ≤ 12 Å) across all predicted binding partners, and the cLIR (contact LIR) percentage, which further restricts to direct intermolecular contacts (Cβ-Cβ ≤ 8 Å).

**Figure 3.**
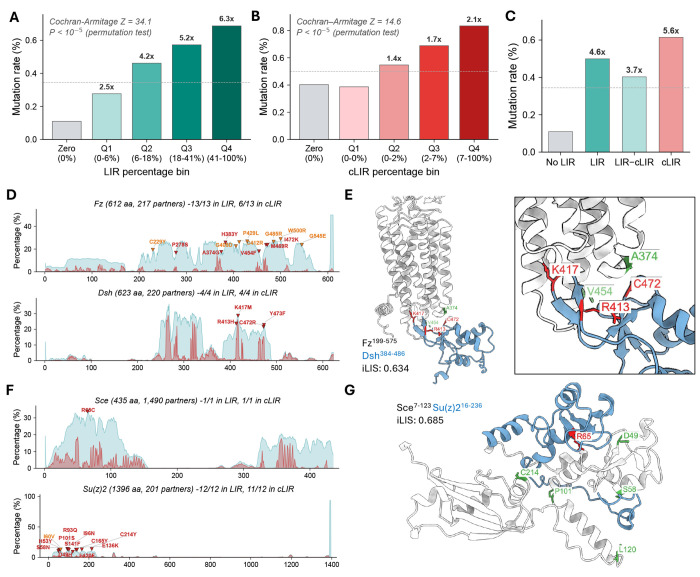
Missense alleles are enriched at predicted interaction interfaces. **(A)** Mutation rate across LIR frequency bins for 801,876 residues in 995 genes. Residues are binned into Zero (never in any interaction domain) and quartiles Q1–Q4 of the non-zero distribution. Fold-enrichment relative to Zero is indicated above each bar. Cochran-Armitage trend test Z = 34.1 (permutation P < 10^−5^). Dashed lines in panels A–C indicate the average mutation rate. **(B)** Mutation rate across cLIR frequency bins for residues within predicted interaction domains (LIR > 0). Cochran-Armitage trend test Z = 14.6 (permutation P < 10^−5^). **(C)** Mutation rate across residue categories: No LIR (not in any interaction domain), LIR (in interaction domain), LIR–cLIR (in interaction domain but not at contact), and cLIR (direct contact). Both domain context and contact interface contribute to mutation enrichment. **(D)** LIR (teal) and cLIR (salmon) frequency profiles for Frizzled (Fz; top) and Dishevelled (Dsh; bottom). Each profile shows the fraction of predicted interacting partners in which a given residue is part of the interaction domain (LIR) or at direct contact (cLIR). Red triangles indicate mapped missense mutations. For Fz, all 13 mutations fall within the interaction domain. For Dsh, all 4 mutations fall at direct contact residues. **(E)** Predicted wild-type Fz–Dsh complex (iLIS = 0.634). Mutation positions in Dsh (red) and Fz (green) are highlighted at the binding interface. **(F)** LIR/cLIR profiles for Sce (top) and Su(z)2 (bottom). The single Sce mutation (R65C) falls at a direct contact residue. Of 12 mapped Su(z)2 mutations, 11 fall at direct contact residues. **(G)** Predicted wild-type Sce–Su(z)2 complex (iLIS = 0.685). Mutation positions in Sce (red) and Su(z)2 (green) are highlighted at the binding interface.

We tested whether mutation rate increases with LIR percentage across these 995 genes. The mutation rate increased monotonically from 0.11% for residues never in any interaction domain to 0.69% for the highest quartile, a 6.3-fold enrichment (Figure 3A). Among residues within an interaction domain, there was additional enrichment at the direct contact interface. The mutation rate increased from 0.40% to 0.83%, a 2.1-fold enrichment (Figure 3B). Within-gene permutation tests confirmed that both trends reflect residue-level specificity rather than gene-level ascertainment bias (P < 10^−5^ for both; Figure S3.1A–B).

To test whether the broader interaction domain also contributes independently of direct contact, we separated domain residues into those making direct contact (cLIR) and those in the domain but not at the contact interface (LIR-cLIR). Compared to non-interacting residues (0.11%), all interaction-domain residues showed elevated mutation rates (LIR: 0.50%; Figure 3C). Within these, domain residues lacking direct contact (LIR-cLIR: 0.40%) were intermediate between non-interacting and direct contact residues (cLIR: 0.61%), indicating that both the domain context and the contact interface contribute to functional constraint. Even among domain-only residues (LIR > 0, cLIR = 0), mutation rate increased monotonically with LIR percentage (2.8–4.7× enrichment; permutation P < 10^−5^; Figure S3.1C–D). Overall, 87% of mapped mutations fell within a predicted interaction domain and 49% at direct contact residues.

Evolutionarily conserved residues tend to produce phenotypes when mutated, raising the possibility that the observed enrichment reflects conservation rather than interface-specific constraint. To test this, we controlled for evolutionary conservation using PhyloP scores (Pollard et al., 2010) from the 27-way insect species alignment (23 *Drosophila* species and 4 outgroup insects) available through the UCSC Genome Browser (Kent et al., 2002). Interface residues were indeed more conserved than non-interface residues (mean PhyloP: non-interface 1.92, interface 2.62), and mutated residues were more conserved than non-mutated residues (Figure S3.1E). However, when we stratified residues into four equal groups by conservation level, the interface enrichment persisted across all conservation levels (3.7×–5.8×, with the highest enrichment in intermediate-conservation residues rather than the most conserved; Figure S3.1F). Logistic regression confirmed that cLIR remained a highly significant predictor of mutation status after adjusting for PhyloP (P = 10^−59^), with conservation accounting for only 8% of the interface effect (Figure S3.1G). These results demonstrate that the enrichment reflects the functional importance of interaction interfaces beyond what evolutionary conservation alone can explain.

### Visualizing allele enrichment in well-characterized signaling proteins

To visualize this enrichment in individual proteins, we first examined Wnt/planar cell polarity (PCP) signaling, where the receptor Frizzled (Fz) couples to Dishevelled (Dsh) via the Dsh DEP domain. Among the 13 mapped mutations of Fz, 6 fell at direct contact residues; for Dsh, all 4 mapped mutations were at direct contact residues (Figure 3D). When we mapped these mutations onto the predicted Fz–Dsh complex, 2 Fz mutations (*fz[24]*: A374G; *fz[GL31]*: V454F) and 3 Dsh mutations (*dsh[1]*: K417M; *dsh[A3]*: R413H; *dsh[A21]*: C472R) mapped to the binding interface (Figure 3E). The three Dsh mutations all reside in the DEP domain and have been shown to specifically disrupt PCP signaling (Axelrod et al., 1998; Boutros et al., 1998; Penton et al., 2002), consistent with selective disruption of the Fz–Dsh interface. A recent DVL2-FZD4 cryo-EM structure (PDB: 8WM9; Qian et al., 2024), published after the AFM training data cutoff, closely matches our predicted Fz-Dsh complex (Figure S3.2A), confirming that the predicted interface to which these PCP-specific alleles map is structurally accurate.

As a second example, we examined the PRC1 ubiquitylation module components Sce (Sex combs extra/dRING, the catalytic E3 ligase) and Su(z)2, a paralog of the canonical PRC1 subunit Psc. Of the 12 mapped mutations in Su(z)2, 11 fall at direct contact residues across predicted binding partners, and 5 map specifically to the predicted Sce–Su(z)2 interface. The single Sce mutation (R65C) also maps to this interface (Figures 3F, 3G). The predicted RING-RING interface matches the known Ring1B–BMI1 crystal structures (PDB: 2CKL, Buchwald et al., 2006; PDB: 2H0D, Li et al., 2006) (Figure S3.2B–C). The prediction also suggests contacts extending beyond the crystallized RING domain into the RAWUL domain of Su(z)2, where the C214Y mutation maps (Nguyen et al., 2017). This pattern extends beyond individual genes: LIR/cLIR profiles for 10 additional genes show alleles clustering at predicted interaction hotspots (Figure S3.3), and structural examination of 16 additional complexes across diverse signaling and regulatory contexts confirms that missense alleles map to or near the predicted binding interface in each case (Figure S3.4). Together, these findings indicate that predicted interaction interfaces are enriched for functionally important residues, as independently defined by classical genetics.

### FlyPredictome database and evidence-supported PPI network

**Figure 4.**
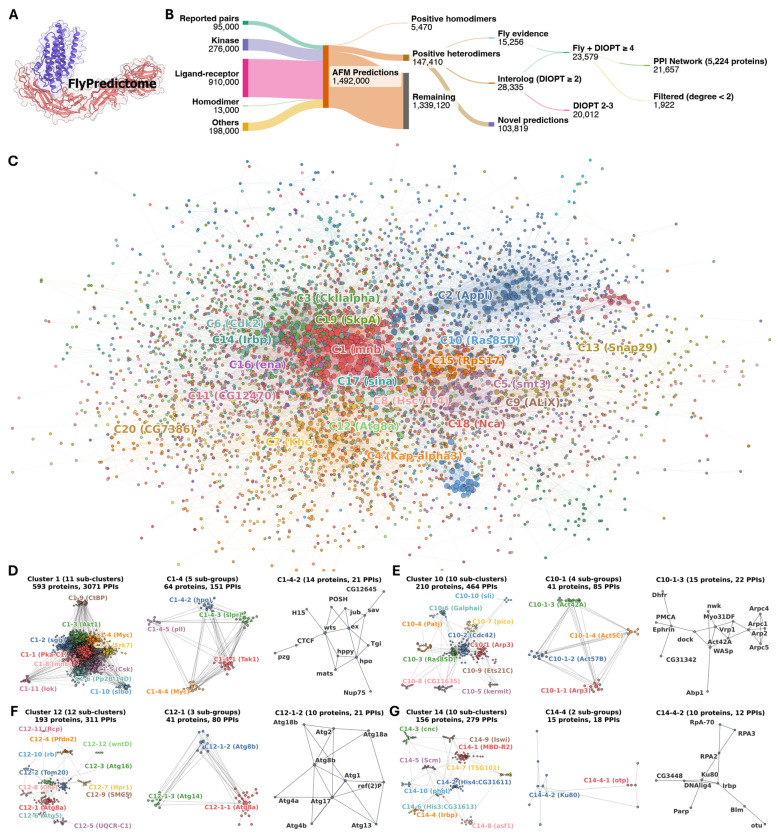
FlyPredictome database and evidence-supported PPI network. **(A)** The FlyPredictome database (https://www.flyrnai.org/tools/fly_predictome). **(B)** Sankey diagram showing the flow from ~1.5 million AFM predictions (sourced from reported PPIs, kinase pairs, ligand–receptor pairs, homodimers, and others) through iLIS threshold classification (iLIS ≥ 0.223; 147,410 positive heterodimers and 5,470 positive homodimers), evidence annotation (fly literature, interolog conservation at DIOPT ≥ 2, or novel), to the final evidence-supported network (fly literature or DIOPT ≥4; 5,224 proteins, 21,657 PPIs), shown in (C). All predictions, including those below threshold, are accessible through FlyPredictome. **(C)** Evidence-supported PPI network colored by cluster assignment. Leiden community detection identified 31 clusters, each annotated with its hub protein. Intra-cluster edges are colored by cluster; inter-cluster edges are gray. An interactive version is available at https://flyark.github.io/FlyPredictome-network. **(D)** Hierarchical decomposition of Cluster 1 (kinases/transcriptional regulators). Left: full cluster (593 proteins, 11 sub-clusters). Center: C1-4 (JNK cascade/growth control; 5 sub-groups). Right: C1-4-2 (14-protein Hippo signaling module). **(E)** Hierarchical decomposition of Cluster 10 (signal transduction/cytoskeleton). Left: full cluster (210 proteins, 10 sub-clusters). Center: C10-1 (Arp2/3 actin nucleation; 4 sub-groups). Right: C10-1-3 (Arp2/3 and actin regulatory module). **(F)** Hierarchical decomposition of Cluster 12 (autophagy). Left: full cluster (193 proteins, 12 sub-clusters). Center: C12-1 (macroautophagy; 3 sub-groups). Right: C12-1-2 (autophagosome assembly module) and C12-1-3 (PI3K nucleation complex). **(G)** Hierarchical decomposition of Cluster 14 (chromatin/DNA repair). Left: full cluster (156 proteins, 10 sub-clusters). Center: C14-4 (NHEJ DNA repair; 2 sub-groups). Right: C14-4-2 (NHEJ ligation complex). Cluster assignments, GO enrichment, network edges, and evidence composition are provided in Tables S3–S7.

With iLIS established as a confidence metric and predicted interfaces validated through allele enrichment, we scaled our predictions to build FlyPredictome, a database for in silico PPI discovery (https://www.flyrnai.org/tools/fly_predictome; Figure 4A). The database contains 1.5 million pairwise AFM predictions, including experimentally reported PPIs (FlyBase, BioGRID, and MIST), kinase interactome predictions, secreted ligand–receptor extracellular domain pairs, and homodimer predictions for nearly all *Drosophila* proteins. After consolidating overlapping prediction sets and isoforms to unique gene pairs, 147,410 heterodimeric PPIs and 5,470 homodimers were classified as positive (iLIS ≥ 0.223; Figure 4B). Among the heterodimeric PPIs, 15,256 are supported by fly literature evidence, 28,335 by interolog conservation (DIOPT score ≥ 2; Hu et al., 2011), and the remaining 103,819 lack prior support, suggesting potentially novel interactions.

To explore the functional organization of the positive PPIs, we selected those supported by fly literature or by high-confidence interologs (DIOPT ≥ 4). Retaining proteins with at least two interactions yielded a network of 5,224 proteins and 21,657 PPIs (Figure 4B). Leiden community detection (Traag et al., 2019) identified 31 clusters (Figure 4C), and GO enrichment analysis confirmed that each cluster corresponds to a distinct biological process (Figure S4.1). To resolve functional heterogeneity within each cluster, we performed recursive sub-clustering (see Methods; Figure S4.2). We highlight four examples below that illustrate how this hierarchical analysis resolves pathway-level modules into individual protein complexes. Full results are available in Tables S3–S7 and the interactive network viewer (https://flyark.github.io/FlyPredictome-network).

In Cluster 1 (kinases and transcriptional regulators), sub-cluster C1–4 (JNK cascade and growth control) contains a 14-protein sub-group (C1-4-2) corresponding to the core Hippo pathway components (the kinases Hpo and Wts, the Wts co-activator Mats, and the scaffold protein Sav) together with upstream regulators Ex and Jub, the Sd co-repressor Tgi, and the MAP4K family kinase Hppy (Figures 4D, S4.3). This C1-4-2 cluster also includes CG12645, a predicted zinc-binding protein localized to late endosomal and lysosomal membranes, which is predicted to bind both Sav (iLIS = 0.535) and the upstream regulator Kibra (iLIS = 0.342), suggesting a potential role in membrane-associated Hippo pathway regulation.

In Cluster 10 (signal transduction and cytoskeleton), sub-cluster C10-1 (Arp2/3-mediated actin nucleation) contains a 15-protein sub-group (C10-1-3) comprising two structurally linked sub-modules: Arp2/3 complex subunits (Arp2, Arpc1, Arpc4, Arpc5) with the WASp actin regulatory module (WASp, Vrp1, Nwk), and Eph-Ephrin signaling components (Ephrin, Dock) (Figures 4E, S4.4). The edges linking these two sub-modules are supported by interolog conservation, consistent with the established role of Eph receptor signaling in driving actin remodeling through N-WASP and Cdc42 (Irie and Yamaguchi, 2002).

In Cluster 12 (autophagy), the macroautophagy sub-cluster C12-1 separates the PI3K complex required for phagophore nucleation (Atg6, Vps15, Pi3K59F, Atg14 in C12-1-3) from a 10-protein module (C12-1-2) containing the Atg1 kinase complex (Atg1, Atg13, Atg17/FIP200), the Atg8 conjugation machinery (Atg4a, Atg4b) and their substrate Atg8b, the PI3P-binding proteins Atg18a and Atg18b, the lipid transfer protein Atg2, and the selective autophagy receptor ref(2)P (p62/SQSTM1 ortholog) (Figures 4F, S4.5), resolving the upstream lipid signaling step from the downstream autophagosome assembly machinery.

In Cluster 14 (chromatin and DNA repair), sub-cluster C14-4 (DNA double-strand break repair) contains a 10-protein sub-group (C14-4-2) comprising the NHEJ factors Ku heterodimer (Irbp and Ku80) and DNA Ligase IV, together with Parp, the RPA complex (RpA-70, RPA2, RPA3), and Blm helicase (Figures 4G, S4.6). This module also includes CG3448, a distant ortholog of human XRCC4, predicted to bind DNAlig4 (iLIS = 0.448). XRCC4 is the obligate partner of DNA Ligase IV in the NHEJ ligation complex across eukaryotes (Sibanda et al., 2001). CG3448 has not been functionally characterized in *Drosophila*, but its co-purification with DNAlig4 in AP-MS (Bhat et al., 2024) and the structural prediction here both support its assignment as the fly XRCC4.

By placing structurally predicted PPIs within a hierarchical network, this approach assigns proteins to functional modules based on their interaction partners, enabling inference of candidate biological roles for uncharacterized proteins. Because the current network is built from interactions supported by existing fly literature or conserved interologs, it is likely to underrepresent pathways for which experimental PPI data are sparse, and assignments based on few edges should be interpreted accordingly. As additional interaction data accumulate, this network will gain both coverage and resolution.

## Discussion

FlyPredictome provides a proteome-scale structural interactome for *Drosophila*. By scoring 1.5 million pairwise AFM predictions with iLIS, we identified positive PPIs across experimentally reported and computationally inferred interaction categories, providing binding interfaces that predict which domains and residues mediate each interaction. Furthermore, by scoring only the confident interaction domain within the full-length proteins, iLIS extends this capability to interactions involving intrinsically disordered regions, which are difficult to detect with global confidence metrics yet mediate many key signaling events in metazoa (van der Lee et al., 2014;Wright and Dyson, 2015). Because iLIS incorporates intermolecular contact information, the resulting predictions are enriched for direct physical associations, enabling construction of an evidence-supported PPI network.

The residue-level detail of these predictions enabled us to demonstrate that phenotype-associated missense mutations are enriched at predicted interaction sites, validating the functional relevance of these structural models. This synergy between genetics and structural prediction is exemplified by the Fz–Dsh interface, where classical fly genetics had identified PCP-specific DEP-domain mutations whose structural consequences are now visualized at residue resolution by AFM predictions. Even for well-characterized signaling pathways where genetic interactions are established, the structural basis of most individual binding events has remained unknown. FlyPredictome provides predicted structural models for many of these interactions.

Beyond *Drosophila*, applying iLIS at our 10% FPR threshold (≥ 0.223) to the recent human pre-dictome dataset (Schmid et al., 2025) recovered 99.5% of their high-confidence pairs, supporting iLIS as a transferable confidence metric across organisms. The 1.6 million pairs were already nominated by KIRC from 200 million possible human pairs based on prior-knowledge features. Thus, iLIS-positive pairs outside SPOC’s strict threshold may represent candidates worth followup validation, particularly for less-characterized proteins where omics-derived features that drive SPOC are sparse. Moreover, because iLIS scores structural confidence directly without requiring pair-level omics features, it can be applied to higher-order assemblies (≥ 3 subunits) and de novo designed binders, settings where the omics-derived features required by classifiers like SPOC are not available.

However, several limitations of the current FlyPredictome dataset should be noted. Our AFM predictions do not account for post-translational modifications, cofactors, or cellular compartmentalization, and are limited to binary interactions. As a result, interactions that depend on these factors or that involve higher-order assemblies may be missed, contributing to the modest recovery rates observed for experimental PPI datasets. Conversely, structurally compatible protein pairs that do not co-localize or co-express in vivo should be interpreted with caution. The iLIS threshold for positive PPI classification may need adjustment for different biological contexts, and different confidence metrics may recover partially overlapping sets of interactions. Continued advances in deep learning-based structure prediction, including improved handling of flexibility, post-translational context, and multimeric assemblies, may recover interactions that current methods miss.

Despite these limitations, FlyPredictome provides all precomputed predictions as an open resource, including those below the positive threshold, rather than reporting only high-confidence interactions. These scores help narrow down candidate interaction partners, while residue-level interface annotations can inform the design of interaction-specific mutations or domain truncations that selectively disrupt individual binding events. The database also provides specialized prediction sets, including kinase interactome and ligand–receptor prediction sets, to serve as a resource for signaling and cell communication studies. The platform will continue to expand as new prediction sets are added. More broadly, the scoring, validation, and allele enrichment framework described here is applicable to any organism with proteome-scale structural predictions and a catalog of phenotype-associated variants.

## Methods

### AlphaFold-Multimer predictions

Protein structure predictions were performed using LocalColabFold v.1.5.5 (Mirdita et al., 2022), which integrates AlphaFold-Multimer v.2.3.1 (Evans et al., 2021). Multiple sequence alignments (MSAs) were generated using colabfold_search against the UniRef30 (version 2202) and mmseqs2/14– 7e284 structural databases. All computations were performed on the Harvard O2 high-performance computing cluster and the Longwood high-performance compute cluster using a variety of NVIDIA GPUs (TeslaV100, RTX6000, RTX8000, A40, A100, A6000, L40S, and H100). Each prediction generated five models with five recycling iterations. Interaction confidence was assessed using the integrated Local Interaction Score (iLIS).

### integrated Local Interaction Score (iLIS)

iLIS is defined as the geometric mean of LIS and cLIS: iLIS = √(LIS × cLIS). LIS (Local Interaction Score) quantifies the confidence of the predicted interaction domain by inversely rescaling interchain PAE values within the confident region (PAE ≤ 12 Å) to a 0-to-1 score, where PAE = 12 Å maps to 0 and PAE = 0 Å maps to 1. However, in some AFM predictions, two protein chains can be positioned in proximity in 3D space without forming genuine intermolecular contacts, resulting in spuriously high LIS values. cLIS (contact-filtered LIS) addresses this by restricting scoring to residue pairs in direct intermolecular contact (Cβ-Cβ ≤ 8 Å within the PAE ≤ 12 Å domain). The geometric mean formulation balances domain-level and contact-level confidence, penalizing predictions where LIS is high but no direct contacts exist (cLIS ≈ 0). Previously, this issue was addressed using a double-cutoff approach requiring both LIS and LIA (Local Interaction Area) thresholds (Kim et al., 2024), an approach that has since been independently adopted (Qi et al., 2026). iLIS simplifies this to a single continuous score. The PAE threshold of 12 Å was selected based on systematic optimization as described in (Kim et al., 2024). The iLIS analysis pipeline is available at https://github.com/flyark/AFM-LIS.

### Other confidence metrics

In addition to iLIS, we benchmarked multiple confidence metrics, grouped into three categories. Local confidence metrics restrict scoring to the high-confidence interaction region: iLIS, LIS (Kim et al., 2024), cLIS, actifpTM (Varga et al., 2025), and ipSAE (Dunbrack, 2025). Global confidence metrics score all inter-chain residue pairs regardless of confidence: ipTM, Model Confidence (Evans et al., 2021), and ifPAE (the mean inter-chain PAE across all residue pairs). Contact-based metrics score residue pairs in direct intermolecular contact without PAE domain restriction: pDockQ (Bryant et al., 2022), pDockQ2 (Zhu et al., 2023), and ifPAE_d8 (mean inter-chain PAE restricted to Cβ-Cβ ≤ 8 Å pairs). For ifPAE and ifPAE_d8, lower values indicate higher-confidence interfaces; inverted values were used in correlation and ROC analyses for consistent directionality. All metrics were evaluated using both the best-ranked model and the average across all five models per pair. Spearman correlation analysis for the benchmark dataset is available in Figure S1.2A–B. Bootstrap confidence intervals and FPR thresholds are provided in Tables S1 and S2, respectively.

### Benchmark dataset construction

The benchmark dataset comprised a positive reference set and multiple control sets. The positive set (774 PPIs) integrates four sources: the human Positive Reference Set (PRS; Braun et al., 2009), the fly PRS from the FlyBi Y2H screen (Tang et al., 2023), the yeast PRS (Yu et al., 2008), and structure-validated interactions from the Eukaryotic Linear Motif (ELM) database (Kumar et al., 2024). The control set (6,004 PPIs) includes Random Reference Sets (RRS) from the three Y2H screens (protein pairs not reported to have an interaction at the time of curation), GFP controls (each PRS protein paired with GFP, serving as a non-interacting folded protein), subcellular compartment controls (PRS proteins paired with the secreted protein Wingless, testing whether compartment mismatch alone produces low scores), and separately shuffled pairs from each PRS and ELM set (pairing proteins with non-cognate partners). A time-split validation set was constructed from heterodimeric protein complexes sourced from the validation dataset of HelixFold-Multimer (Fang et al., 2024), whose experimental structures were deposited in the PDB after the AFM training data cutoff. All pairs were predicted using full-length protein sequences.

### Threshold calibration

Thresholds for positive PPI classification were calibrated using ROC analysis on the Y2H-derived PRS from yeast, fly, and human screens as positives, and three control sets as negatives: RRS, PRS proteins paired with GFP, and PRS proteins paired with Wingless (Wg, a secreted protein serving as a subcellular compartment control). For each metric, the threshold was set at 10% false positive rate (FPR = 0.10), i.e., the score at which 10% of control pairs exceed the threshold. We used Y2H-derived PRS as positives and RRS as negatives because inclusion of ELM interactions or randomly shuffled pairs shifted the threshold substantially, resulting in the loss of many well-established PPIs. Thresholds were computed at multiple FPR levels (1%, 5%, 10%) across pLDDT subgroups (0–50, 50–70, 70–100) and the total set, for both best-model and average-model scoring (Table S2). For downstream classification, we adopted the best-model total iLIS threshold at 10% FPR (≥ 0.223) and ipTM (≥ 0.48). These thresholds may require adjustment for specific biological contexts; for example, weaker or more transient interactions may benefit from a more permissive threshold, while high-confidence applications may warrant stricter cutoffs.

### Evaluation against *Drosophila* experimental PPI datasets

Predictions were evaluated against four experimental datasets: FlyBi Y2H interactions (Tang et al., 2023), DPiM AP-MS interactions (Guruharsha et al., 2011), DPiM2 AP-MS interactions (Bhat et al., 2024), and literature-curated PPIs from FlyBase (Öztürk-Çolak et al., 2024), BioGRID (Oughtred et al., 2021), and MIST (Hu et al., 2018). Recovery rate was defined as the fraction of experimentally reported interactions scoring above the iLIS threshold.

### Comparison with computational PPI prediction datasets

For comparison with Peng and Zhao (2026), we obtained Supporting Information S1 Appendix (DOI: 10.1371/journal.pcbi.1013899.s011), which reports pair identifiers, pDockQ, and iLIS for 27,711 AlphaFold-Multimer predictions on *Drosophila* protein pairs derived from STRING ≥ 0.9 confidence. Pairs were canonicalized to FBgn-FBgn pairs and isoform-level duplicates collapsed by maximum score per pair (27,683 unique pairs). Pairs called positive by iLIS (≥ 0.223) but with pDockQ < 0.23, corresponding to the “possible” tier defined by Peng and Zhao (pDockQ < 0.23 with ipTM ≥ 50 or minimum inter-chain PAE ≤ 10 Å), were tabulated as PPIs uniquely captured by iLIS. All 1,070 such pairs satisfied the minimum PAE ≤ 10 Å criterion (100%), indicating that the iLIS rescue is driven primarily by recognition of confident interaction domains via inter-chain PAE.

For analysis of the Schmid et al. (2025) human predictome, iLIS was computed on the complete set of 1,614,047 AlphaFold-Multimer predictions (KIRC-nominated). Positive calls were defined at iLIS_best ≥ 0.223 (10% FPR). SPOC scores for the same pairs were obtained from the predic-tomes.org S3 deposit (https://predictomes-hsbps-dataset.s3.us-east-1.amazonaws.com/20251110_hs_predictome_pair_scores.csv.gz). To assess concordance with the SPOC classifier (Schmid and Walter, 2025), we compared iLIS positive calls to SPOC ≥ 0.86, the threshold reported by Schmid et al. (2025) for high-confidence calls (10% FDR).

### Missense allele enrichment analysis

Missense mutations were compiled from two FlyBase sources (Öztürk-Çolak et al., 2024): allele descriptions and genetic interaction records. Mutations were mapped to AFM-predicted structural models using exact position matching or Needleman-Wunsch alignment-based (Needleman and Wunsch, 1970) isoform correction when necessary. Because isoform differences and annotation discrepancies can shift residue numbering, only mutations whose reference amino acid matched the AFM model at the mapped position were retained, yielding 2,761 unique gene-position pairs across 995 genes.

For each residue in a protein, two interaction frequency metrics were computed across all prediction models with at least one intermolecular contact: the LIR percentage (fraction of models in which the residue falls within a confident interaction domain, PAE ≤ 12 Å) and the cLIR percentage (fraction of models in which the residue makes direct intermolecular contact, Cβ-Cβ ≤ 8 Å). Residues were binned into five groups: Zero (never in any interaction domain), and quartiles Q1–Q4 of the nonzero LIR distribution.

The Cochran-Armitage trend test was used to assess monotonic enrichment of mutation rate across LIR/cLIR bins. Within-gene permutation tests (100,000 permutations) shuffled mutation positions within each gene independently to control for gene-level ascertainment bias. Evolutionary conservation was assessed using PhyloP scores (Pollard et al., 2010) from the 27-way insect species alignment (23 *Drosophila* species and 4 outgroup insects) available through the UCSC Genome Browser (Kent et al., 2002). Logistic regression was performed with mutation status as the dependent variable and cLIR and PhyloP as independent variables.

### PPI network construction and clustering

The evidence-supported direct PPI network was constructed by selecting positive predictions (iLIS ≥ 0.223) supported by either fly literature evidence (physical and genetic interactions) or conserved interactions in other species (DIOPT ortholog score ≥ 4; Hu et al., 2011). Proteins with fewer than two connections were removed, and the giant connected component was extracted.

Community detection was performed using the Leiden algorithm (Traag et al., 2019; leidenalg Python package, RBConfigurationVertexPartition, resolution = 1.1, seed = 42), identifying 31 clusters. Sub-clustering was performed on each main cluster independently using the same Leiden algorithm with per-cluster resolution optimization: for each cluster, resolutions from 0.5 to 4.0 (step 0.1) were tested, and the resolution yielding the highest modularity was selected. The significance of each split was assessed by a permutation test: node-to-community assignments were shuffled 100 times while preserving community sizes, and the split was accepted only if the observed modularity exceeded the 95th percentile of the null distribution (p < 0.05). This produced 257 sub-clusters. Recursive sub-sub-clustering further decomposed sub-clusters using the same permutation-based criterion (minimum 5 genes), yielding 407 sub-sub-clusters.

Gene Ontology enrichment analysis was performed using the hypergeometric test with FlyBase GO annotations (GAF release 2026-03-28), querying GO:BP, GO:MF, and GO:CC ata significance threshold of 0.05 (BH correction). Redundant terms were removed by Lin semantic similarity (cutoff 0.7). Full cluster assignments, GO enrichment, network edges, and evidence composition are provided in Tables S3–S7. An interactive network viewer is available at https://flyark.github.io/FlyPredictome-network.

### Visualization

Data visualization and statistical plots were generated using Python (matplotlib, seaborn, matplotlib-venn) and R. Network visualization used igraph and NetworkX. Sankey diagrams were generated using SankeyMATIC (https://sankeymatic.com). Protein structure visualization was performed using UCSF ChimeraX (Pettersen et al., 2021) and Mol* (Sehnal et al., 2021) via LIVIA (Local Interaction Visualization and Analysis; https://flyark.github.io/LIVIA) (Kim and Perrimon, 2026), a browser-based tool for interface confidence scoring and 3D visualization. Structural alignment of the predicted *Drosophila* Fz-Dsh complex with the human DVL2-FZD4 cryo-EM structure (PDB: 8WM9; Qian et al., 2024) was performed using the matchmaker command in ChimeraX. RMSD values were calculated for the confident interaction region (LIR residues) and for the contact interface residues (cLIR residues) separately.

### FlyPredictome database implementation

The FlyPredictome database (https://www.flyrnai.org/tools/fly_predictome) was implemented as a website built with the Symfony framework and hosted on a traditional LAMP stack, with underlying data stored in a MySQL database. FlyPredictome uses the HTML5 canvas element for drawing heatmaps, Mol* (Sehnal et al., 2021) for protein visualization, Vega-Lite for certain plots, and generates SVG files for Pearson correlation graphs. The backend is written in PHP, while frontend views are rendered with the Twig templating engine. jQuery was used for DOM manipulation, DataTables was integrated for displaying tabular data with search functionality, and Bootstrap was employed for layout, formatting, and styling. Icons throughout the interface are provided by the Font Awesome library. Both the web application and its underlying database are hosted on the O2 high-performance computing cluster at Harvard Medical School, maintained by the Research Computing group.

## Supplementary Material

Supplement 1

2

## Data Availability

The FlyPredictome database, including iLIS values and AlphaFold-Multimer models for all 1.5 million predicted PPIs, is available for interactive exploration at the FlyPredictome portal (https://www.flyrnai.org/tools/fly_predictome). The interactive viewer for the evidence-supported PPI network is available at https://flyark.github.io/FlyPredictome-network. iLIS source code is available at https://github.com/flyark/AFM-LIS. Full benchmark data, threshold calibration tables, network clustering results, and GO enrichment analyses are provided in Tables S1–S7. All positive PPI predictions with confidence scores are provided in Table S8. iLIS-positive pairs from the Schmid et al. (2025) human predictome (227,714 pairs at iLIS_best ≥ 0.223), with paired SPOC and KIRC scores, are provided in Table S9.
