# Supplementary material for: FlyPredictome: A structural atlas of predicted protein-protein interactions in Drosophila
Source: bioRxiv. 2026 May 11:2026.04.14.718529. Originally published 2026 Apr 16. Preprint. [Version 2] doi: 10.64898/2026.04.14.718529 (PMC13104844; doi:10.64898/2026.04.14.718529)
Supplement: 2 [file NIHPP2026.04.14.718529v2-supplement-2.pdf]

bioRxiv preprint doi: https://doi.org/10.64898/2026.04.14.718529; this version posted May 11, 2026. The copyright holder for this preprint (which was not certified by peer review) is the author/funder, who has granted bioRxiv a license to display the preprint in perpetuity. It is made available under aCC-BY 4.0 International license.

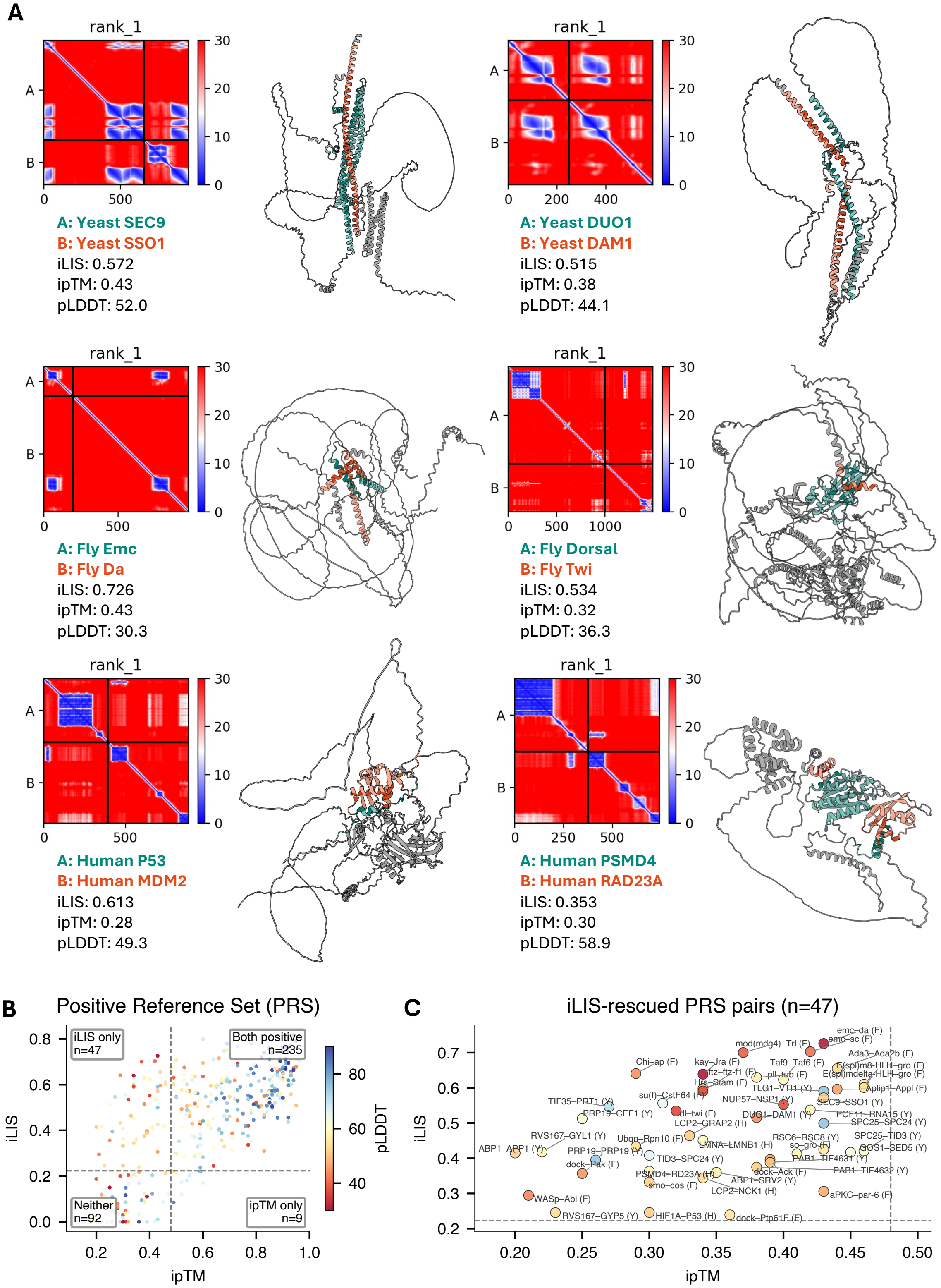

Figure S1.1. iLIS rescues well-established PPIs with low ipTM scores. (A) Six representative Positive Reference Set (PRS) pairs from yeast (SEC9–SSO1, DUO1–DAM1), fly (Emc–Da,

bioRxiv preprint doi: https://doi.org/10.64898/2026.04.14.718529; this version posted May 11, 2026. The copyright holder for this preprint (which was not certified by peer review) is the author/funder, who has granted bioRxiv a license to display the preprint in perpetuity. It is made available under aCC-BY 4.0 International license.

Dorsal–Twi), and human (p53–MDM2, PSMD4–RAD23A) correctly identified by integrated Local Interaction Score (iLIS) but missed by ipTM. For each pair, the Predicted Aligned Error (PAE) heatmap (left) and AFM-predicted structure (right) are shown. PAE measures the expected positional error between residue pairs; low values (blue) indicate high-confidence relative positioning. In the structures, Local Interaction Residue (LIR; PAE ≤ 12 Å) regions are colored in teal (chain A) and orange (chain B), with darker shading indicating contact LIR (cLIR; PAE ≤ 12 Å and Cβ-Cβ ≤ 8 Å); non-confident regions are shown in gray. Despite low ipTM scores (0.28–0.43) and low pLDDT (30.3–58.9), all six complexes exhibit clearly defined local interfaces, visible as blue off-diagonal blocks in the PAE heatmaps, with high iLIS (0.353–0.726). **(B)** Scatter plot of iLIS versus ipTM for all PRS pairs, colored by pLDDT. The iLIS-only quadrant (top left, n=47) is enriched for low-pLDDT pairs. **(C)** Zoomed view of the 47 iLIS-rescued PRS pairs with individual annotations. Species indicated as (F) fly, (Y) yeast, (H) human.

bioRxiv preprint doi: https://doi.org/10.64898/2026.04.14.718529; this version posted May 11, 2026. The copyright holder for this preprint (which was not certified by peer review) is the author/funder, who has granted bioRxiv a license to display the preprint in perpetuity. It is made available under aCC-BY 4.0 International license.

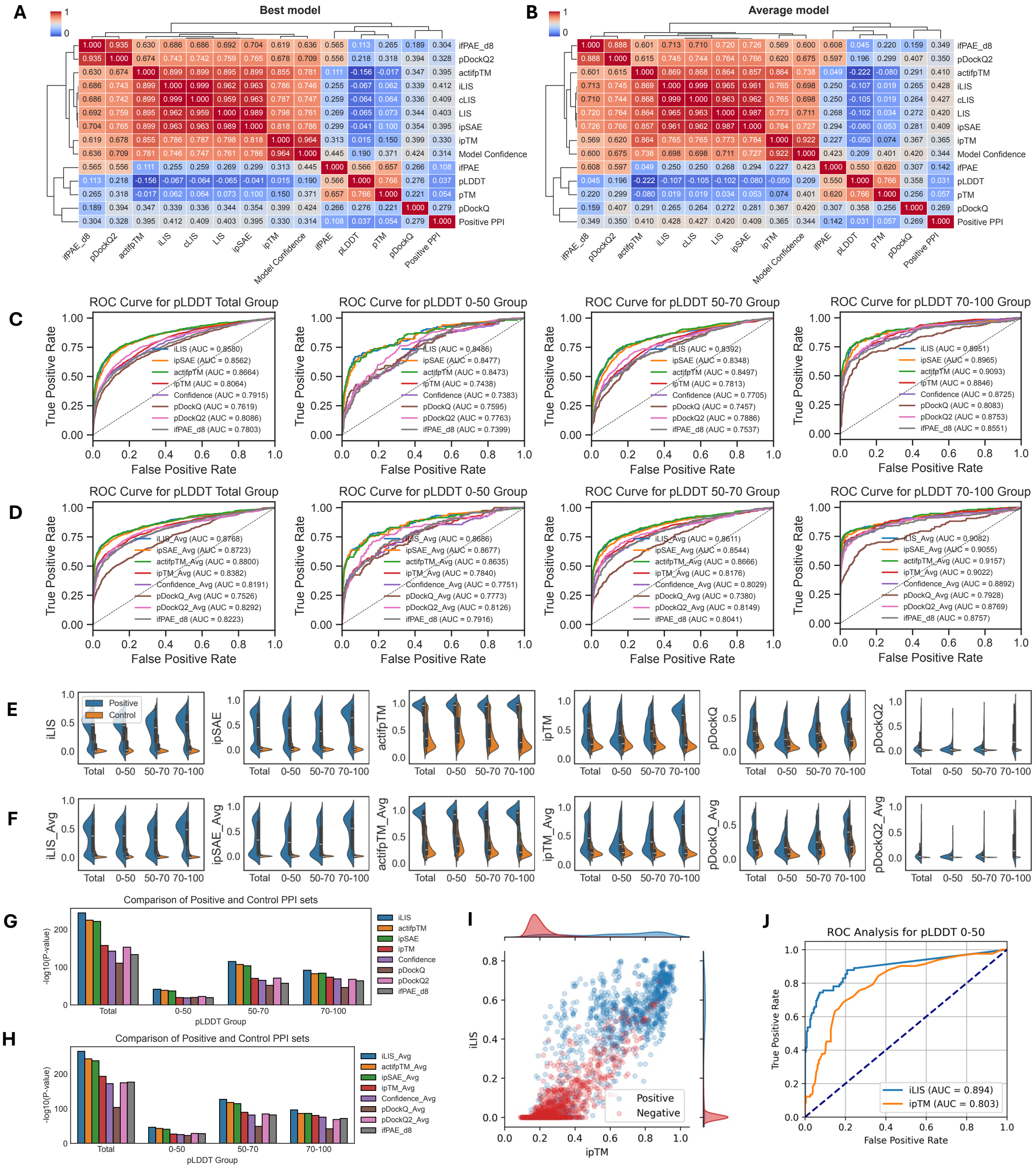

Figure S1.2. Comprehensive benchmarking of confidence metrics. (A–B) Spearman correlation clustermaps among all confidence metrics for (A) best-model and (B) average-model (averaged across all 5 ranks) scoring, with hierarchical clustering. Local confidence metrics (iLIS, LIS, cLIS, actifpTM, ipSAE) cluster together, and iLIS showed the highest correlation with positive PPI. Note: ifPAE and ifPAE_d8 are inverted so that higher values indicate better predictions. **(C–D)** ROC curves across pLDDT subgroups for (C) best-model and (D) average-model scoring.

bioRxiv preprint doi: https://doi.org/10.64898/2026.04.14.718529; this version posted May 11, 2026. The copyright holder for this preprint (which was not certified by peer review) is the author/funder, who has granted bioRxiv a license to display the preprint in perpetuity. It is made available under aCC-BY 4.0 International license.

Local confidence metrics outperform other metrics across all subgroups. **(E–F)** Score distributions for positive (blue) and control (orange) PPIs across pLDDT groups for (E) best-model and (F) average-model metrics. **(G–H)** Statistical separation (-log10 P-value, Mann-Whitney U test) between positive and control PPIs for each metric across pLDDT subgroups. **(I)** iLIS versus ipTM scatter plot on the time-split benchmark (sourced from Fang et al., 2024; structures deposited after AFM training cutoff). **(J)** ROC analysis comparing iLIS and ipTM performance on the low-pLDDT subset (0–50) of the time-split benchmark.

bioRxiv preprint doi: https://doi.org/10.64898/2026.04.14.718529; this version posted May 11, 2026. The copyright holder for this preprint (which was not certified by peer review) is the author/funder, who has granted bioRxiv a license to display the preprint in perpetuity. It is made available under aCC-BY 4.0 International license.

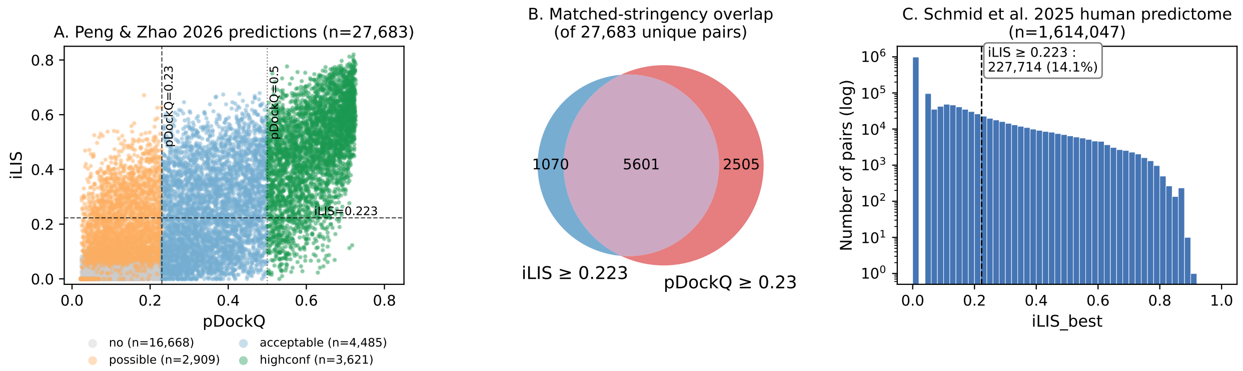

Figure S1.3. Benchmarking iLIS against two recent computational PPI prediction datasets. (A) Per-pair iLIS versus pDockQ across 27,683 *Drosophila* AlphaFold-Multimer predictions from Peng and Zhao (2026), color-coded by their four-tier classification ("no", "possible", "acceptable", "highconf"). Dashed lines: iLIS positive threshold (≥ 0.223), Peng and Zhao "acceptable" threshold (pDockQ ≥ 0.23), and "high confidence" threshold (pDockQ ≥ 0.5). **(B)** Matched-stringency overlap of positive calls on the Peng and Zhao set (iLIS ≥ 0.223 vs pDockQ ≥ 0.23): 5,601 pairs called positive by both, 1,070 unique to iLIS, 2,505 unique to pDockQ. **(C)** iLIS distribution across the 1,614,047 human protein pairs of the recent human predictome (Schmid et al., 2025); 227,714 pairs (14.1%) meet iLIS ≥ 0.223 (dashed line).

bioRxiv preprint doi: https://doi.org/10.64898/2026.04.14.718529; this version posted May 11, 2026. The copyright holder for this preprint (which was not certified by peer review) is the author/funder, who has granted bioRxiv a license to display the preprint in perpetuity. It is made available under aCC-BY 4.0 International license.

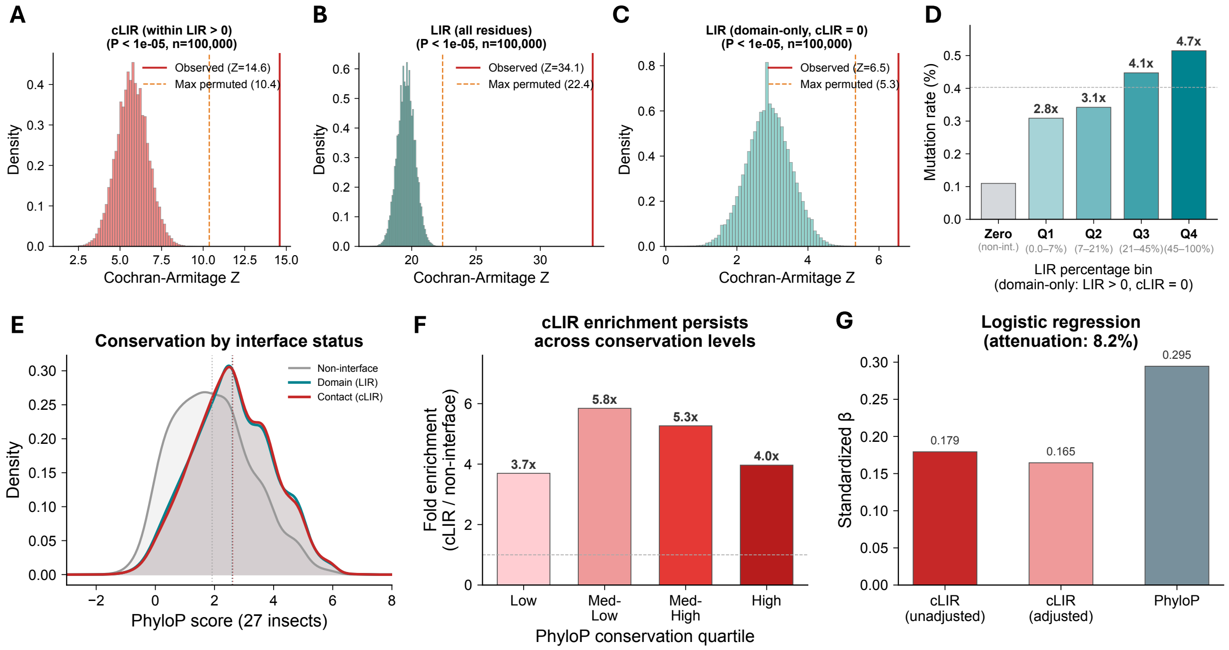

Figure S3.1. Permutation tests and evolutionary conservation controls for allele enrichment. (A–C) Within-gene permutation tests (100,000 permutations). Observed Cochran-Armitage trend test Z (red solid line) versus distribution of permuted Z values (orange dashed line indicates maximum permuted Z). (A) cLIR among residues within interaction domains (LIR > 0; observed Z = 14.6, max permuted = 10.4). (B) LIR across all residues (observed Z = 34.1, max permuted = 22.4). (C) LIR among domain-only residues (cLIR = 0; observed Z = 6.5, max permuted = 5.3). In all three tests, the observed Z exceeds the maximum permuted value ($P < 10^{-5}$). **(D)** LIR enrichment among domain-only residues (LIR > 0, cLIR = 0), showing that even non-contact domain residues are enriched for mutations (2.8–4.7× fold enrichment). Dashed line indicates average mutation rate for domain-only residues. **(E)** PhyloP conservation score distributions for non-interface, domain (LIR), and contact (cLIR) residues. **(F)** cLIR enrichment (fold over non-interface) stratified by PhyloP conservation quartile. Interface enrichment persists across all conservation levels (3.7–5.8×). **(G)** Logistic regression standardized β coefficients for cLIR (unadjusted and PhyloP-adjusted) and PhyloP alone. Conservation accounts for only 8.2% attenuation of the interface effect.

bioRxiv preprint doi: https://doi.org/10.64898/2026.04.14.718529; this version posted May 11, 2026. The copyright holder for this preprint (which was not certified by peer review) is the author/funder, who has granted bioRxiv a license to display the preprint in perpetuity. It is made available under aCC-BY 4.0 International license.

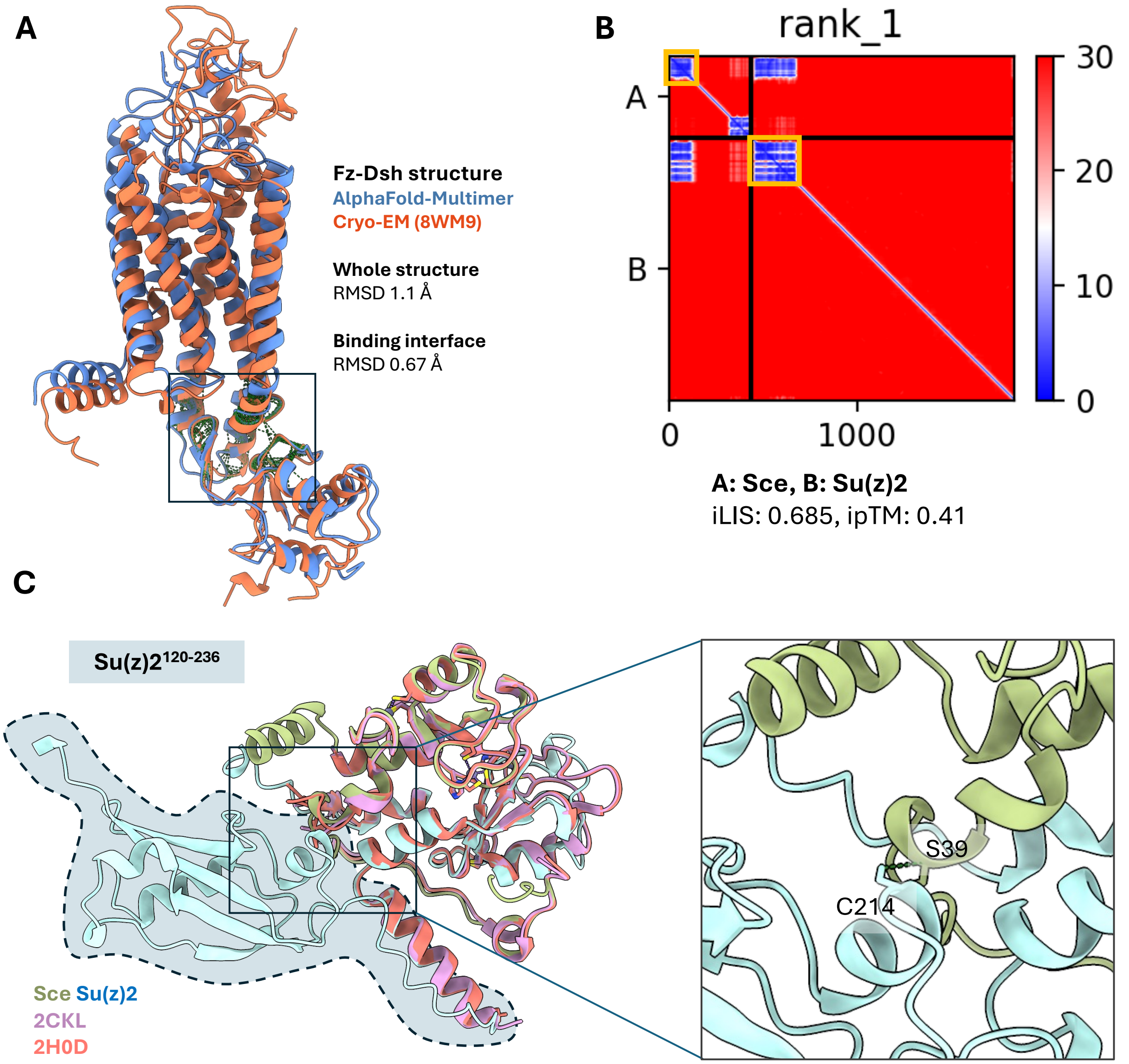

Figure S3.2. Structural comparison of predicted complexes with experimental structures. **(A)** Superposition of the predicted *Drosophila* Fz–Dsh complex (blue) onto the human DVL2–FZD4 cryo-EM structure (PDB: 8WM9; Qian et al., 2024; orange). RMSD = 1.1 Å across the overall structure; contact interface RMSD = 0.67 Å. Box highlights the DEP domain–receptor interface region. **(B)** PAE heatmap of the predicted Sce–Su(z)2 complex. Orange box indicates the interaction domain shown in (C). **(C)** Superposition of the predicted *Drosophila* Sce–Su(z)2 complex (region highlighted in B) onto the human Ring1B–BMI1 crystal structures (PDB: 2CKL, Buchwald et al., 2006; PDB: 2H0D, Li et al., 2006). The blue shaded area with dashed outline indicates the region of Su(z)2 encompassing the RAWUL domain (residues 120-228), not resolved in existing Ring1B–BMI1 structures. Inset highlights the mutation site C214 (Nguyen et al., 2017) in Su(z)2, positioned in contact with Sce S39 within the predicted interface.

bioRxiv preprint doi: https://doi.org/10.64898/2026.04.14.718529; this version posted May 11, 2026. The copyright holder for this preprint (which was not certified by peer review) is the author/funder, who has granted bioRxiv a license to display the preprint in perpetuity. It is made available under aCC-BY 4.0 International license.

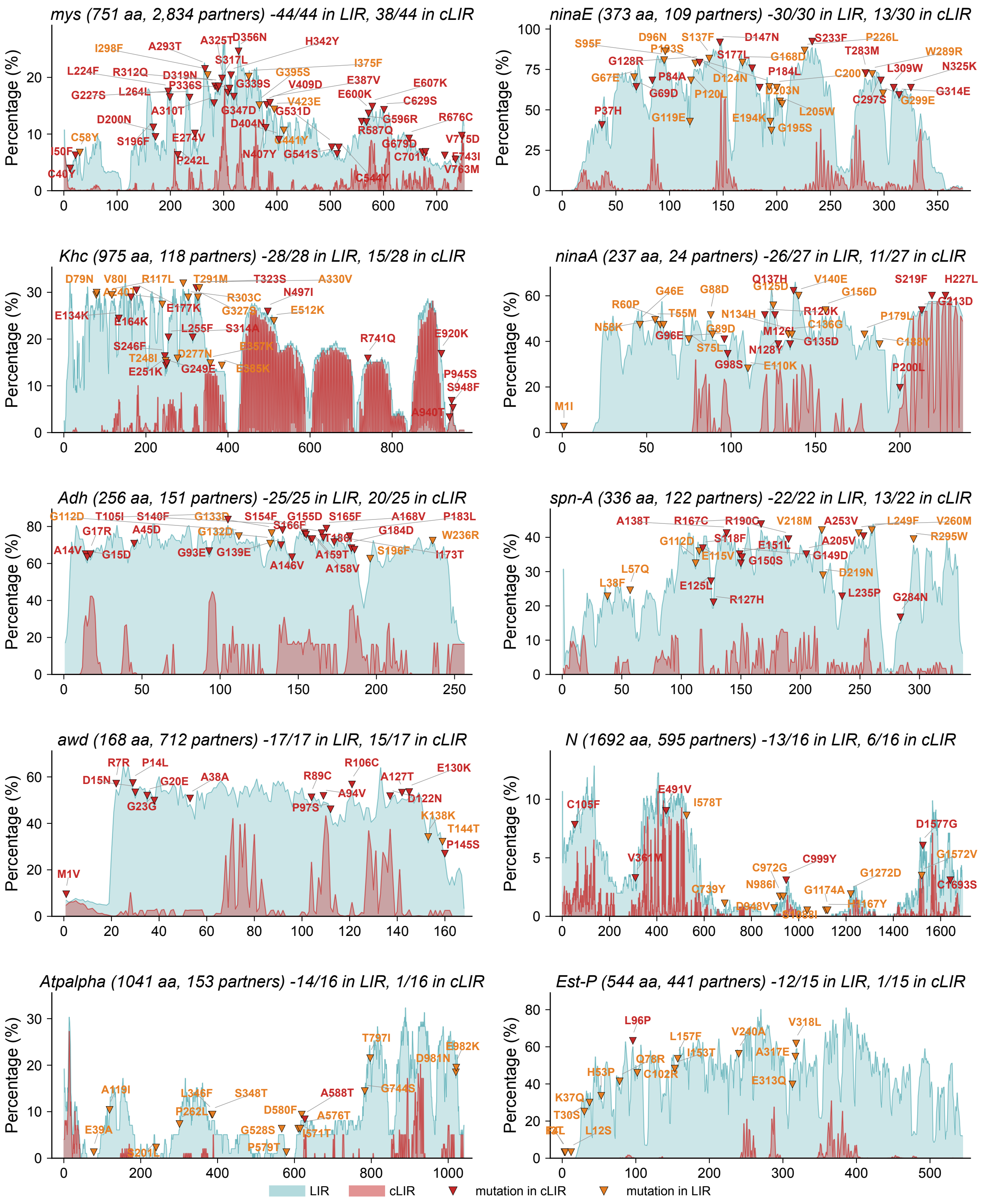

Figure S3.3. LIR/cLIR profiles for 10 example genes with multiple mapped mutations. LIR (teal) and cLIR (salmon) frequency profiles. Each profile shows the fraction of predicted interacting partners in which a given residue is part of the interaction domain (LIR) or at direct contact

bioRxiv preprint doi: https://doi.org/10.64898/2026.04.14.718529; this version posted May 11, 2026. The copyright holder for this preprint (which was not certified by peer review) is the author/funder, who has granted bioRxiv a license to display the preprint in perpetuity. It is made available under aCC-BY 4.0 International license.

(cLIR). Red triangles indicate mapped missense mutations. Missense alleles consistently cluster at predicted interaction hotspots across diverse genes.

bioRxiv preprint doi: https://doi.org/10.64898/2026.04.14.718529; this version posted May 11, 2026. The copyright holder for this preprint (which was not certified by peer review) is the author/funder, who has granted bioRxiv a license to display the preprint in perpetuity. It is made available under aCC-BY 4.0 International license.

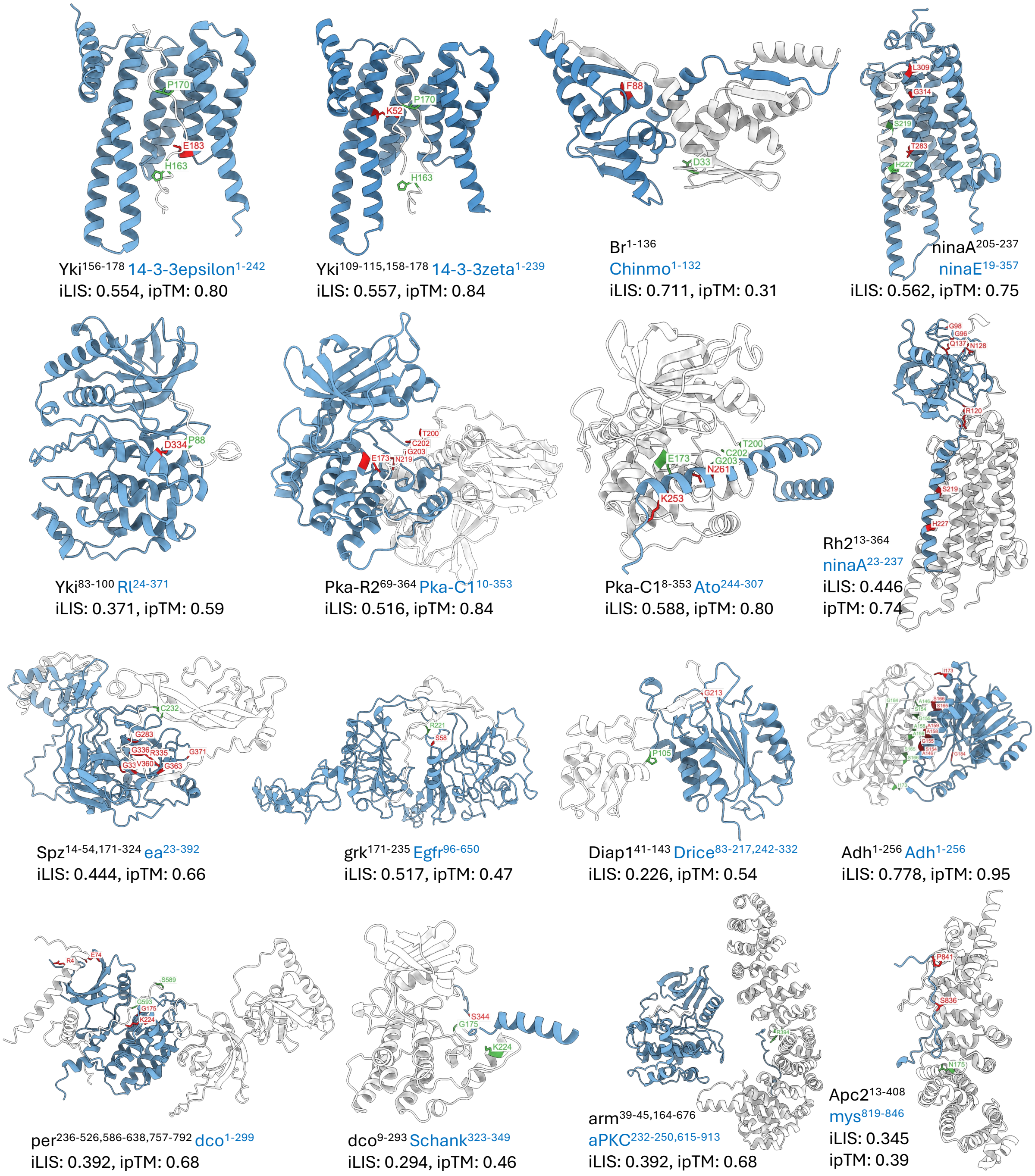

Figure S3.4. Structural examination of missense alleles at predicted interaction interfaces. Sixteen predicted complexes spanning Hippo, Toll, RTK, Wnt, apoptotic signaling, chromatin regulation, circadian rhythm, and cell adhesion. For each complex, missense alleles (red/green) are mapped onto the predicted structure, confirming localization at the binding interface.

bioRxiv preprint doi: https://doi.org/10.64898/2026.04.14.718529; this version posted May 11, 2026. The copyright holder for this preprint (which was not certified by peer review) is the author/funder, who has granted bioRxiv a license to display the preprint in perpetuity. It is made available under aCC-BY 4.0 International license.

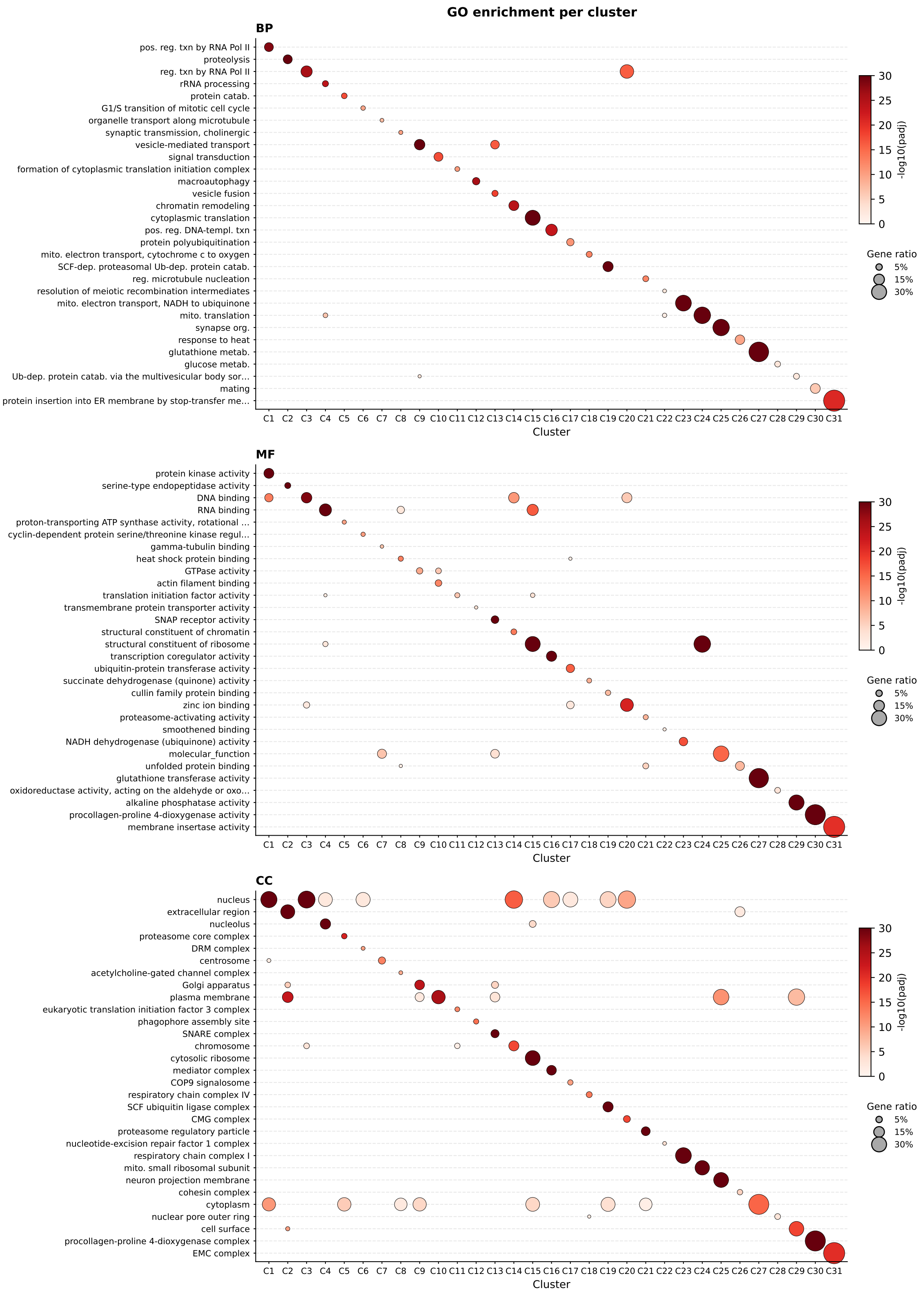

Figure S4.1. GO enrichment per cluster: top terms. Dot plots showing the top enriched GO terms (Biological Process [BP], Molecular Function [MF], Cellular Component [CC]) for all 31 clus-

bioRxiv preprint doi: https://doi.org/10.64898/2026.04.14.718529; this version posted May 11, 2026. The copyright holder for this preprint (which was not certified by peer review) is the author/funder, who has granted bioRxiv a license to display the preprint in perpetuity. It is made available under aCC-BY 4.0 International license.

ters. Dot size indicates gene ratio; dot color indicates adjusted p-value (red = most significant). Enrichment was performed using hypergeometric test with FlyBase GO annotations (BH correction, padj < 0.05). Redundant terms were removed by Lin semantic similarity (cutoff 0.7).

bioRxiv preprint doi: https://doi.org/10.64898/2026.04.14.718529; this version posted May 11, 2026. The copyright holder for this preprint (which was not certified by peer review) is the author/funder, who has granted bioRxiv a license to display the preprint in perpetuity. It is made available under aCC-BY 4.0 International license.

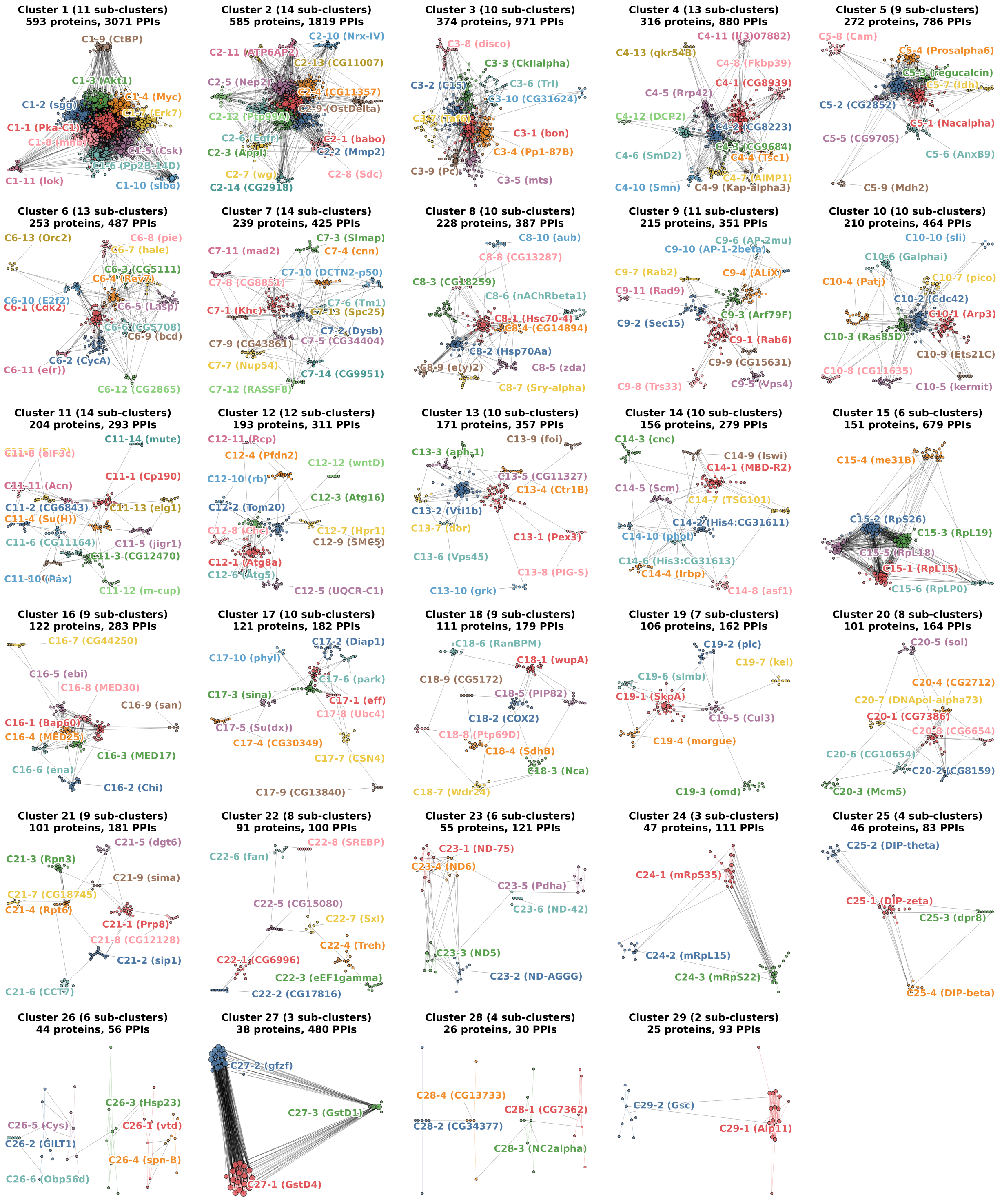

Figure S4.2. Sub-cluster network views for all clusters. Each cluster is shown with sub-clusters in distinct colors. The most connected hub gene in each sub-cluster is labeled.

bioRxiv preprint doi: https://doi.org/10.64898/2026.04.14.718529; this version posted May 11, 2026. The copyright holder for this preprint (which was not certified by peer review) is the author/funder, who has granted bioRxiv a license to display the preprint in perpetuity. It is made available under aCC-BY 4.0 International license.

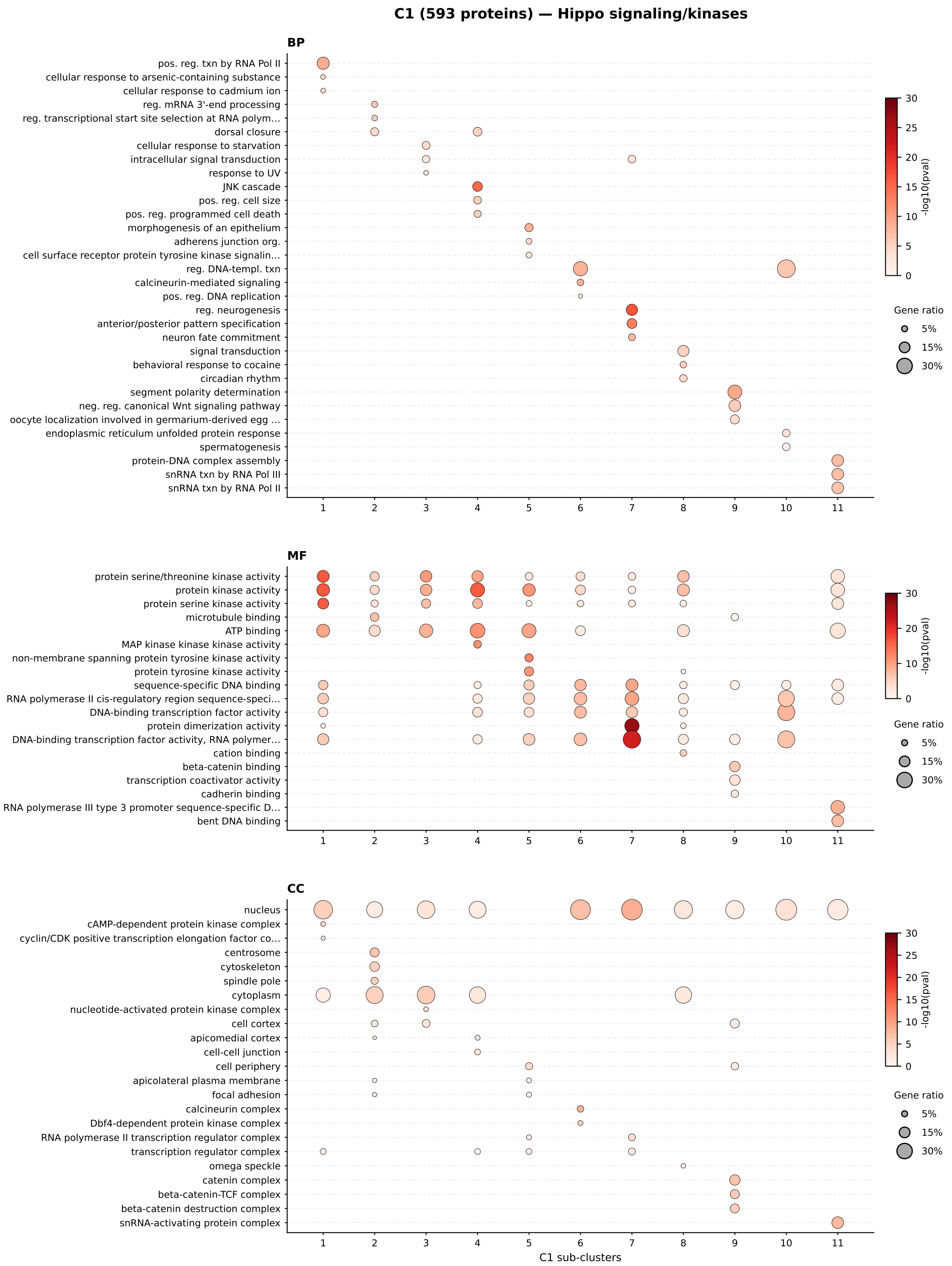

Figure S4.3. Sub-cluster GO enrichment for Cluster 1 (Hippo signaling/kinases). Dot plots showing the top enriched GO terms per sub-cluster for BP, MF, and CC.

bioRxiv preprint doi: https://doi.org/10.64898/2026.04.14.718529; this version posted May 11, 2026. The copyright holder for this preprint (which was not certified by peer review) is the author/funder, who has granted bioRxiv a license to display the preprint in perpetuity. It is made available under aCC-BY 4.0 International license.

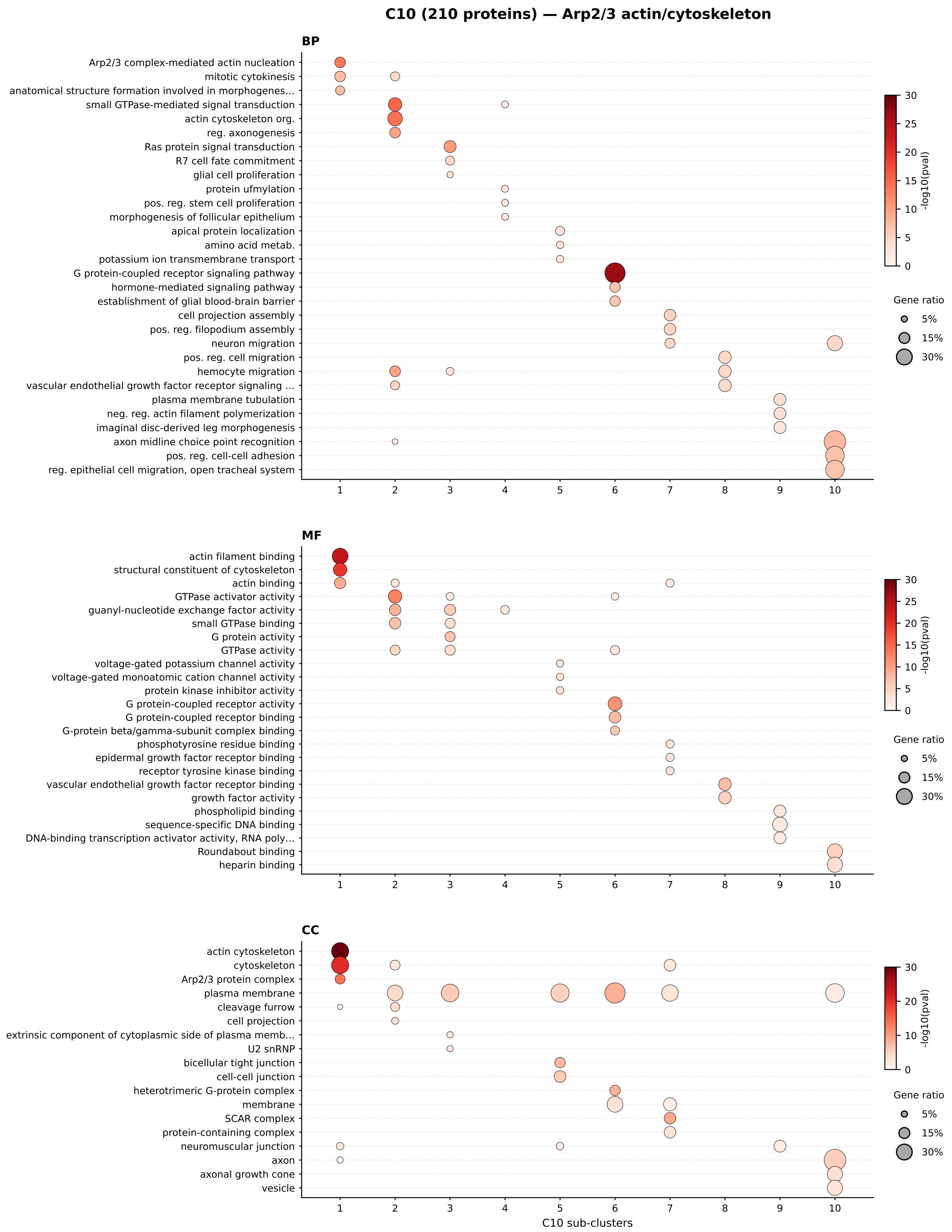

Figure S4.4. Sub-cluster GO enrichment for Cluster 10 (Arp2/3 actin/cytoskeleton). Dot plots showing the top enriched GO terms per sub-cluster for BP, MF, and CC.

bioRxiv preprint doi: https://doi.org/10.64898/2026.04.14.718529; this version posted May 11, 2026. The copyright holder for this preprint (which was not certified by peer review) is the author/funder, who has granted bioRxiv a license to display the preprint in perpetuity. It is made available under aCC-BY 4.0 International license.

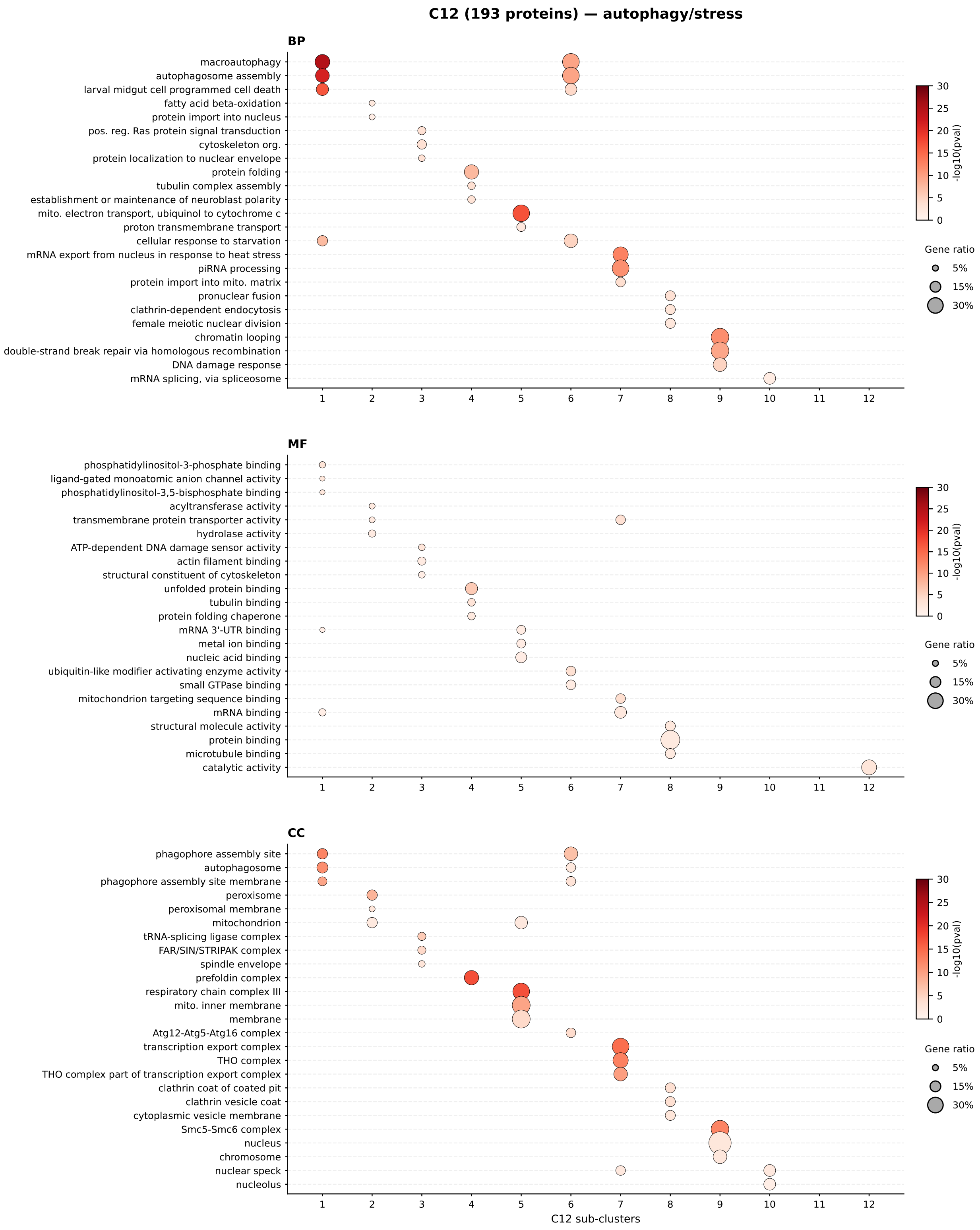

Figure S4.5. Sub-cluster GO enrichment for Cluster 12 (autophagy/stress). Dot plots showing the top enriched GO terms per sub-cluster for BP, MF, and CC.

bioRxiv preprint doi: https://doi.org/10.64898/2026.04.14.718529; this version posted May 11, 2026. The copyright holder for this preprint (which was not certified by peer review) is the author/funder, who has granted bioRxiv a license to display the preprint in perpetuity. It is made available under aCC-BY 4.0 International license.

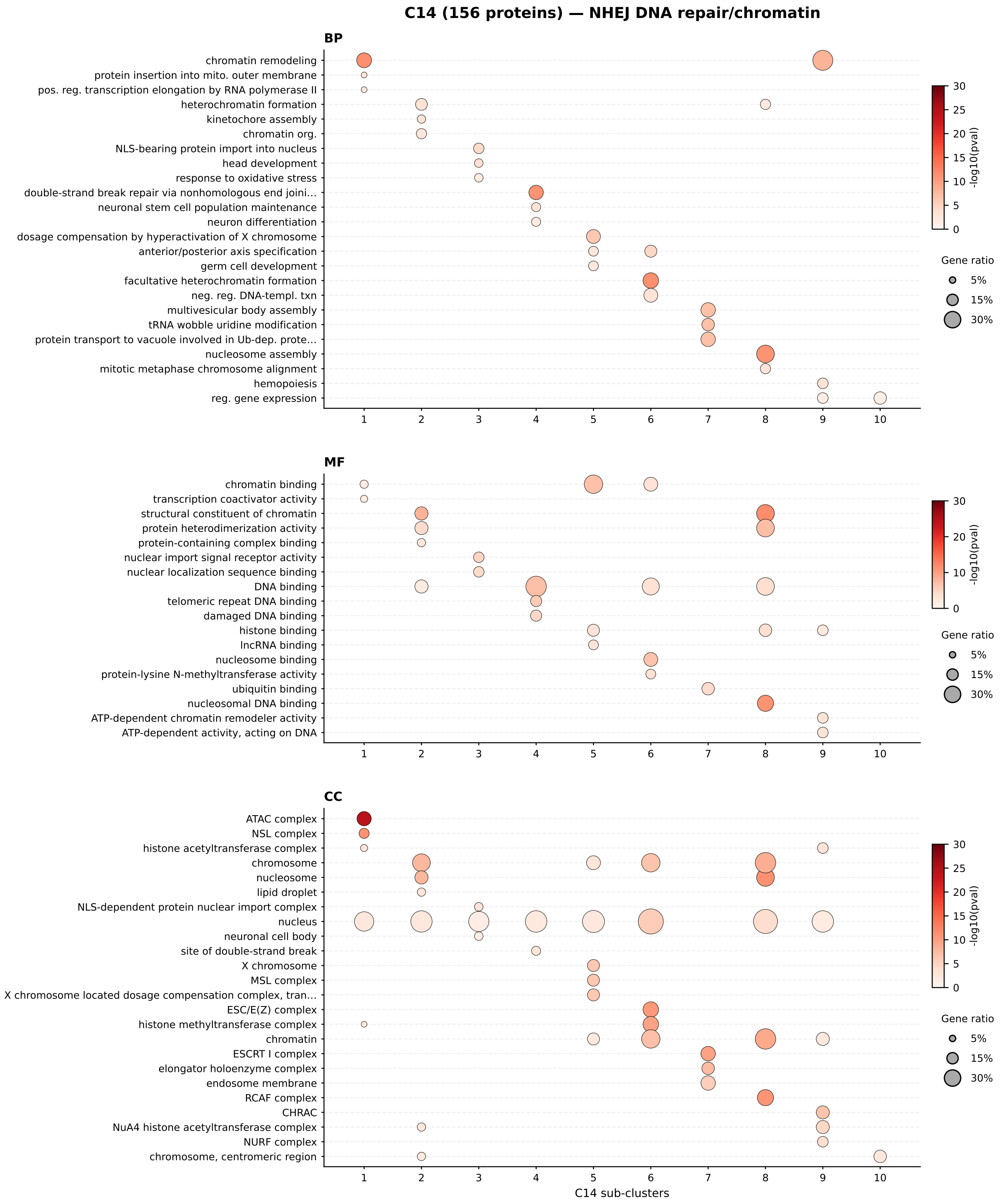

Figure S4.6. Sub-cluster GO enrichment for Cluster 14 (NHEJ DNA repair/chromatin). Dot plots showing the top enriched GO terms per sub-cluster for BP, MF, and CC.
